# Supplementary material for: Power analysis for cluster randomized trials with continuous coprimary endpoints
Source: Biometrics. Author manuscript; Available in PMC 2024 Aug 13. (PMC11321238; doi:10.1111/biom.13692)
Supplement: Appendix [file NIHMS2012065-supplement-Appendix.pdf]

Supporting Information for "Power analysis for cluster randomized trials with continuous co-primary endpoints" by Yang et al.

Web Appendix A: EM algorithm for MLMM

Resuming the notation in the main text, we write y_{ijk} as the kth ($k = 1, \ldots, K$) continuous endpoint for the jth ($j = 1, \ldots, m_i$) subject in the ith ($i = 1, \ldots, n$) cluster. The outcome model in matrix notation for each subject is,

$$\boldsymbol{y}_{ij} = \boldsymbol{x}_{ij}^T \boldsymbol{\xi} + \boldsymbol{\phi}_i + \boldsymbol{e}_{ij}, \tag{1}$$

where $\boldsymbol{y}_{ij} = (y_{ij1}, \ldots, y_{ijK})^T$ is the collection of K endpoints, $\boldsymbol{x}_{ij} = (\boldsymbol{I}_K, \boldsymbol{I}_K)$ is the $K \times 2K$ design matrix, $\boldsymbol{\xi} = (\gamma_1, \ldots, \gamma_K, \beta_1, \ldots, \beta_K)^T$ are the collection of all regression coefficients, $\boldsymbol{\phi}_i \sim \mathcal{N}(\boldsymbol{0}, \boldsymbol{\Sigma}_{\boldsymbol{\phi}})$, $\boldsymbol{e}_{ij} \sim \mathcal{N}(\boldsymbol{0}, \boldsymbol{\Sigma}_{\boldsymbol{e}})$ are the cluster-level random intercept and the error vector. Let $\boldsymbol{\theta} = (\boldsymbol{\xi}, \text{vec}(\boldsymbol{\Sigma}_{\boldsymbol{\phi}}), \text{vec}(\boldsymbol{\Sigma}_{\boldsymbol{e}}))^T$ be the vector of unknown parameters in the multivariate linear mixed model in (1), then the complete data log-likelihood is

$$\begin{aligned} l(\boldsymbol{\theta}) =& \text{const} - \frac{\sum_{i=1}^n m_i}{2} \log |\boldsymbol{\Sigma}_e| - \frac{n}{2} \log |\boldsymbol{\Sigma}_\phi| - \frac{1}{2} \sum_{i=1}^n \boldsymbol{\phi}_i^T \boldsymbol{\Sigma}_\phi^{-1} \boldsymbol{\phi}_i - \\ & \frac{1}{2} \sum_{i=1}^n \sum_{j=1}^{m_i} (\boldsymbol{y}_{ij} - \boldsymbol{x}_{ij}^T \boldsymbol{\xi} - \boldsymbol{\phi}_i)^T \boldsymbol{\Sigma}_e^{-1} (\boldsymbol{y}_{ij} - \boldsymbol{x}_{ij}^T \boldsymbol{\xi} - \boldsymbol{\phi}_i), \end{aligned}$$

where 'const' stands for a constant that is free of model parameters.

Expectation step (E-step): Define $Q(\boldsymbol{\theta}|\widehat{\boldsymbol{\theta}}^{(t)}) = E\left[l(\boldsymbol{\theta})|\widehat{\boldsymbol{\theta}}^{(t)}\right]$ as the expected value of the log-likelihood function given the current estimates/updates $\widehat{\boldsymbol{\theta}}^{(t)}$, and $\boldsymbol{y}_i = (\boldsymbol{y}_{i1}^T, \ldots, \boldsymbol{y}_{i,m_i}^T)^T$ be the vector of all co-primary endpoints in cluster i, applying the properties of the multivariate normal distribution gives

$$\begin{aligned} \mathbb{E}(\boldsymbol{\phi}_i|\boldsymbol{y}_i, \boldsymbol{\theta}^{(t)}) &= \left(\boldsymbol{\Sigma}_{\boldsymbol{\phi}}^{-1} + m_i \boldsymbol{\Sigma}_{\boldsymbol{e}}^{-1}\right)^{-1} \boldsymbol{\Sigma}_{\boldsymbol{e}}^{-1} \sum_{j=1}^{m_i} (\boldsymbol{y}_{ij} - \boldsymbol{x}_{ij}^T \boldsymbol{\xi}) \\ \text{var}(\boldsymbol{\phi}_i|\boldsymbol{y}_i, \boldsymbol{\theta}^{(t)}) &= \left(\boldsymbol{\Sigma}_{\boldsymbol{\phi}}^{-1} + m_i \boldsymbol{\Sigma}_{\boldsymbol{e}}^{-1}\right)^{-1} \\ \mathbb{E}(\boldsymbol{\phi}_i \boldsymbol{\phi}_i^T|\boldsymbol{y}_i, \boldsymbol{\theta}^{(t)}) &= \text{var}(\boldsymbol{\phi}_i|\boldsymbol{y}_i, \boldsymbol{\theta}^{(t)}) + \mathbb{E}(\boldsymbol{\phi}_i|\boldsymbol{y}_i, \boldsymbol{\theta}^{(t)}) \mathbb{E}(\boldsymbol{\phi}_i^T|\boldsymbol{y}_i, \boldsymbol{\theta}^{(t)}) \end{aligned}$$

Maximization step (M-step): For iteration $t+1$, we compute $\widehat{\boldsymbol{\theta}}^{(t+1)}$ by maximizing

the quantity $Q(\boldsymbol{\theta}|\widehat{\boldsymbol{\theta}}^{(t)})$. Taking the partial derivatitives of $Q(\boldsymbol{\theta}|\widehat{\boldsymbol{\theta}}^{(t)})$ yields:

$$\frac{\partial Q(\boldsymbol{\theta}|\widehat{\boldsymbol{\theta}}^{(t)})}{\partial \boldsymbol{\xi}} = \sum_{i=1}^{n}\sum_{j=1}^{m_i} \boldsymbol{x}_{ij}\boldsymbol{\Sigma}_{\boldsymbol{e}}^{-1}\boldsymbol{x}_{ij}^T\boldsymbol{\xi} - \sum_{i=1}^{n}\sum_{j=1}^{m_i} \boldsymbol{x}_{ij}\boldsymbol{\Sigma}_{\boldsymbol{e}}^{-1}\left\{\boldsymbol{y}_{ij} - \mathbb{E}(\boldsymbol{\phi}_i|\boldsymbol{y}_i, \boldsymbol{\theta}^{(t)})\right\} = 0 \tag{2}$$

Solving (2) gives

$$\widehat{\boldsymbol{\xi}}^{(t+1)} = \left\{\sum_{i=1}^{n}\sum_{j=1}^{m_i} \boldsymbol{x}_{ij}\boldsymbol{\Sigma}_{\boldsymbol{e}}^{-1}\boldsymbol{x}_{ij}^T\right\}^{-1}\left\{\sum_{i=1}^{n}\sum_{j=1}^{m_i} \boldsymbol{x}_{ij}\boldsymbol{\Sigma}_{\boldsymbol{e}}^{-1}\boldsymbol{y}_{ij} - \sum_{i=1}^{n}\sum_{j=1}^{m_i} \boldsymbol{x}_{ij}\boldsymbol{\Sigma}_{\boldsymbol{e}}^{-1}\mathbb{E}(\boldsymbol{\phi}_i|\boldsymbol{y}_i, \boldsymbol{\theta}^{(t)})\right\}$$

Similarly, differentiating with respect to $\boldsymbol{\Sigma}_{\phi}^{-1}$ obtains

$$\frac{\partial Q(\boldsymbol{\theta}|\widehat{\boldsymbol{\theta}}^{(t)})}{\partial (\boldsymbol{\Sigma}_{\boldsymbol{\phi}}^{-1})} = \frac{n}{2}\boldsymbol{\Sigma}_{\boldsymbol{\phi}} - \frac{1}{2}\sum_{i=1}^{n}\mathbb{E}(\boldsymbol{\phi}_i\boldsymbol{\phi}_i^T|\boldsymbol{y}_i, \boldsymbol{\theta}^{(t)}) = 0,$$

which implies

$$\widehat{\boldsymbol{\Sigma}}_{\boldsymbol{\phi}}^{(t+1)} = \frac{1}{n}\sum_{i=1}^{n}\mathbb{E}(\boldsymbol{\phi}_i\boldsymbol{\phi}_i^T|\boldsymbol{y}_i, \boldsymbol{\theta}^{(t)}).$$

Differentiating with respect to $\boldsymbol{\Sigma}_{\boldsymbol{e}}^{-1}$ gives

$$\frac{\partial Q(\boldsymbol{\theta}|\widehat{\boldsymbol{\theta}}^{(t)})}{\partial (\boldsymbol{\Sigma}_{\boldsymbol{e}}^{-1})} = \frac{\sum_{i=1}^{n} m_i}{2}\boldsymbol{\Sigma}_e - \frac{1}{2}\sum_{i=1}^{n}\sum_{j=1}^{m_i}\left\{\boldsymbol{y}_{ij} - \boldsymbol{x}_{ij}^T\boldsymbol{\xi} - \mathbb{E}(\boldsymbol{\phi}_i|\boldsymbol{y}_i, \boldsymbol{\theta}^{(t)})\right\}\left\{\boldsymbol{y}_{ij} - \boldsymbol{x}_{ij}^T\boldsymbol{\xi} - \mathbb{E}(\boldsymbol{\phi}_i|\boldsymbol{y}_i, \boldsymbol{\theta}^{(t)})\right\}^T = 0$$

which implies

$$\begin{aligned}
\widehat{\boldsymbol{\Sigma}}_{\boldsymbol{e}}^{(t+1)} &= \frac{1}{\sum_{i=1}^{n} m_i}\sum_{i=1}^{n}\sum_{j=1}^{m_i}\left(\boldsymbol{y}_{ij} - \boldsymbol{x}_{ij}^T\boldsymbol{\xi} - \mathbb{E}(\boldsymbol{\phi}_i|\boldsymbol{y}_i, \boldsymbol{\theta}^{(t)})\right)\left(\boldsymbol{y}_{ij} - \boldsymbol{x}_{ij}^T\boldsymbol{\xi} - \mathbb{E}(\boldsymbol{\phi}_i|\boldsymbol{y}_i, \boldsymbol{\theta}^{(t)})\right)^T \\
&= \frac{1}{\sum_{i=1}^{n} m_i}\Bigg\{\sum_{i=1}^{n}\sum_{j=1}^{m_i}(\boldsymbol{y}_{ij} - \boldsymbol{x}_{ij}^T\boldsymbol{\xi})(\boldsymbol{y}_{ij} - \boldsymbol{x}_{ij}^T\boldsymbol{\xi})^T + \sum_{i=1}^{n} m_i\mathbb{E}(\boldsymbol{\phi}_i\boldsymbol{\phi}_i^T|\boldsymbol{y}_i, \boldsymbol{\theta}^{(t)}) - \\
&\qquad \sum_{i=1}^{n}\sum_{j=1}^{m_i}\mathbb{E}(\boldsymbol{\phi}_i|\boldsymbol{y}_i, \boldsymbol{\theta}^{(t)})(\boldsymbol{y}_{ij} - \boldsymbol{x}_{ij}^T\boldsymbol{\xi})^T + \sum_{i=1}^{n}\sum_{j=1}^{m_i}(\boldsymbol{y}_{ij} - \boldsymbol{x}_{ij}^T\boldsymbol{\xi})\mathbb{E}(\boldsymbol{\phi}_i^T|\boldsymbol{y}_i, \boldsymbol{\theta}^{(t)})\Bigg\}.
\end{aligned}$$

To obtain the point estimates for $\boldsymbol{\theta}$, we will iterate between the E-step and the M-step until

the increment of the log-likelihood, $|l(\widehat{\boldsymbol{\theta}}^{(t+1)}) - l(\widehat{\boldsymbol{\theta}}^{(t)})|$, is within a pres-specified convergence threshold Δ (say, 0.0001). The standard error estimates can then be obtained by inverting the hessian matrix of the observed log-likelihood $l(\boldsymbol{\theta})$ evaluated at the maximum likelihood estimates. We provide the R code to operationalize the EM algorithm for MLMM in the first author's GitHub at https://github.com/siyunyang/coprimary_CRT.

Web Appendix B: Technical details for Theorem 1

Recall that the Feasible Generalized Least Square (FGLS) estimator is denoted by $\widehat{\boldsymbol{\theta}} = \left(\sum_{i=1}^n \boldsymbol{W}_i^T \boldsymbol{V}_i^{-1} \boldsymbol{W}_i\right)^{-1} \left(\sum_{i=1}^n \boldsymbol{W}_i^T \boldsymbol{V}_i^{-1} \boldsymbol{y}_i\right)$, where

$$\begin{aligned} \boldsymbol{U}_n = \sum_{i=1}^n \boldsymbol{W}_{\boldsymbol{i}}^T \boldsymbol{V}_{\boldsymbol{i}}^{-1} \boldsymbol{W}_{\boldsymbol{i}} &= \sum_{i=1}^n \boldsymbol{W}_{\boldsymbol{i}}^T \left\{ \boldsymbol{I}_m \otimes \boldsymbol{\Sigma}_{\boldsymbol{e}}^{-1} + \boldsymbol{J}_m \otimes \frac{1}{m} \left[(\boldsymbol{\Sigma}_{\boldsymbol{e}} + m\boldsymbol{\Sigma}_{\boldsymbol{\phi}})^{-1} - \boldsymbol{\Sigma}_{\boldsymbol{e}}^{-1} \right] \right\} \boldsymbol{W}_{\boldsymbol{i}} \\ &= \begin{pmatrix} nm & 0 \\ 0 & \sum_{i=1}^n (z_i - \bar{z})^2 m \end{pmatrix} (\boldsymbol{\Sigma}_{\boldsymbol{e}} + m\boldsymbol{\Sigma}_{\boldsymbol{\phi}})^{-1}, \end{aligned} \tag{3}$$

and

$$\left(\sum_{i=1}^n \boldsymbol{W}_i^T \boldsymbol{V}_i^{-1} \boldsymbol{y}_i \right) =$$

$$\sum_{i=1}^n \left[\mathbf{1}_m^T \otimes \begin{pmatrix} \boldsymbol{I}_K \\ \boldsymbol{I}_K (z_i - \bar{z}) \end{pmatrix} \right] \times \left[\boldsymbol{I}_m \otimes \boldsymbol{\Sigma}_{\boldsymbol{e}}^{-1} + \boldsymbol{J}_m \otimes \frac{1}{m} \left\{ (\boldsymbol{\Sigma}_{\boldsymbol{e}} + m\boldsymbol{\Sigma}_{\boldsymbol{\phi}})^{-1} - \boldsymbol{\Sigma}_{\boldsymbol{e}}^{-1} \right\} \right] \times \begin{pmatrix} \boldsymbol{y}_{i1} \\ \vdots \\ \boldsymbol{y}_{im} \end{pmatrix}. \tag{4}$$

The upper block of (4) is

$$\begin{aligned} \text{upper block of (4)} &= \sum_{i=1}^n \left[\mathbf{1}_m^T \otimes \boldsymbol{\Sigma}_{\boldsymbol{e}}^{-1} + \mathbf{1}_m^T \otimes \frac{1}{m} \left\{ (\boldsymbol{\Sigma}_{\boldsymbol{e}} + m\boldsymbol{\Sigma}_{\boldsymbol{\phi}})^{-1} - \boldsymbol{\Sigma}_{\boldsymbol{e}}^{-1} \right\} \right] \times \begin{pmatrix} \boldsymbol{y}_{i1} \\ \vdots \\ \boldsymbol{y}_{im} \end{pmatrix} \\ &= \sum_{i=1}^n \left[\mathbf{1}_m^T \otimes (\boldsymbol{\Sigma}_{\boldsymbol{e}} + m\boldsymbol{\Sigma}_{\boldsymbol{\phi}})^{-1} \right] \times \begin{pmatrix} \boldsymbol{y}_{i1} \\ \vdots \\ \boldsymbol{y}_{im} \end{pmatrix} = \sum_{i=1}^n \sum_{j=1}^m (\boldsymbol{\Sigma}_{\boldsymbol{e}} + m\boldsymbol{\Sigma}_{\boldsymbol{\phi}})^{-1} \boldsymbol{y}_{ij}, \end{aligned}$$

and the lower block of (4) is

$$\text{lower block of (4)} = \sum_{i=1}^{n} \left[\mathbf{1}_m^T \otimes \boldsymbol{\Sigma}_{\boldsymbol{e}}^{-1}(z_i - \bar{z}) + \mathbf{1}_m^T \otimes \frac{1}{m} \left\{ (\boldsymbol{\Sigma}_{\boldsymbol{e}} + m\boldsymbol{\Sigma}_{\boldsymbol{\phi}})^{-1} - \boldsymbol{\Sigma}_{\boldsymbol{e}}^{-1} \right\} (z_i - \bar{z}) \right] \times \begin{pmatrix} \boldsymbol{y}_{i1} \\ \vdots \\ \boldsymbol{y}_{im} \end{pmatrix}$$

$$= \sum_{i=1}^{n} (z_i - \bar{z}) \left[\mathbf{1}_m^T \otimes (\boldsymbol{\Sigma}_{\boldsymbol{e}} + m\boldsymbol{\Sigma}_{\boldsymbol{\phi}})^{-1} \right] \times \begin{pmatrix} \boldsymbol{y}_{i1} \\ \vdots \\ \boldsymbol{y}_{im} \end{pmatrix} = \sum_{i=1}^{n} \sum_{j=1}^{m} (z_i - \bar{z}) (\boldsymbol{\Sigma}_{\boldsymbol{e}} + m\boldsymbol{\Sigma}_{\boldsymbol{\phi}})^{-1} \boldsymbol{y}_{ij}.$$

It follows that the FGLS estimator for $\boldsymbol{\theta}$ is

$$\widehat{\boldsymbol{\theta}} = \boldsymbol{U}_n^{-1} \times \begin{pmatrix} \sum_{i=1}^{n} \sum_{j=1}^{m} (\boldsymbol{\Sigma}_{\boldsymbol{e}} + m\boldsymbol{\Sigma}_{\boldsymbol{\phi}})^{-1} \boldsymbol{y}_{ij} \\ \sum_{i=1}^{n} \sum_{j=1}^{m} (z_i - \bar{z}) (\boldsymbol{\Sigma}_{\boldsymbol{e}} + m\boldsymbol{\Sigma}_{\boldsymbol{\phi}})^{-1} \boldsymbol{y}_{ij} \end{pmatrix}$$

$$= \begin{pmatrix} (nm)^{-1} & 0 \\ 0 & \sum_{i=1}^{n} m^{-1} (z_i - \bar{z})^{-2} \end{pmatrix} (\boldsymbol{\Sigma}_{\boldsymbol{e}} + m\boldsymbol{\Sigma}_{\boldsymbol{\phi}}) \times \begin{pmatrix} \sum_{i=1}^{n} \sum_{j=1}^{m} (\boldsymbol{\Sigma}_{\boldsymbol{e}} + m\boldsymbol{\Sigma}_{\boldsymbol{\phi}})^{-1} \boldsymbol{y}_{ij} \\ \sum_{i=1}^{n} \sum_{j=1}^{m} (z_i - \bar{z}) (\boldsymbol{\Sigma}_{\boldsymbol{e}} + m\boldsymbol{\Sigma}_{\boldsymbol{\phi}})^{-1} \boldsymbol{y}_{ij} \end{pmatrix}$$

$$= \begin{pmatrix} \dfrac{1}{nm} \displaystyle\sum_{i=1}^{n} \sum_{j=1}^{m} \boldsymbol{y}_{ij} \\ \dfrac{\sum_{i=1}^{n} \sum_{j=1}^{m} m^{-1} (z_i - \bar{z}) \boldsymbol{y}_{ij}}{\sum_{i=1}^{n} (z_i - \bar{z})^2} \end{pmatrix},$$

implying that the FGLS estimator for the vector of treatment effect estimators $\widehat{\boldsymbol{\beta}} = (\widehat{\beta}_1, \ldots, \widehat{\beta}_K)^T = \left\{ \sum_{i=1}^{n} (z_i - \bar{z})^2 \right\}^{-1} \left\{ \sum_{i=1}^{n} \sum_{j=1}^{m} m^{-1} (z_i - \bar{z}) \boldsymbol{y}_{ij} \right\}$ and is free of any ICCs.

Next, we derive the limiting distribution for the treatment effect parameters, $\sqrt{n}(\widehat{\boldsymbol{\beta}} - \boldsymbol{\beta})$, which follows the multivariate normal distribution with mean zeros and covariance matrix equal to the lower-right $K \times K$ block of

$$\boldsymbol{\Omega}_{\boldsymbol{\beta}} = \lim_{n \to \infty} n \text{var}(\widehat{\boldsymbol{\theta}}) = \lim_{n \to \infty} \left(n^{-1} \boldsymbol{U}_n \right)^{-1} = \frac{1}{m \sigma_z^2} (\boldsymbol{\Sigma}_{\boldsymbol{e}} + m\boldsymbol{\Sigma}_{\boldsymbol{\phi}}),$$

where $\sigma_z^2 = \bar{z}(1 - \bar{z})$ is the binomial variance of the cluster-level treatment indicator. Based on the one-to-one mappings from the variance component matrices and the three types of

ICCs, the covariance parameters in the joint distribution of $\widehat{\boldsymbol{\beta}}$ are equivalently written as

$$\omega_k^2 = n\text{var}(\widehat{\beta}_k) = \frac{\left(\sigma_{\phi k}^2 + \sigma_{ek}^2\right)\left\{1 + (m-1)\rho_0^k\right\}}{m\sigma_z^2} \tag{5}$$

$$\omega_{kk'} = n\text{cov}(\widehat{\beta}_k, \widehat{\beta}_{k'}) = \frac{\sqrt{\sigma_{\phi k}^2 + \sigma_{ek}^2}\sqrt{\sigma_{\phi k'}^2 + \sigma_{ek'}^2}\left\{\rho_2^{kk'} + (m-1)\rho_1^{kk'}\right\}}{m\sigma_z^2}, \tag{6}$$

for $k = 1, \ldots, K$, and $k' \neq k$. One can further set $\sigma_z^2 = 1/4$ under equal treatment allocation.

Web Appendix C: Technical details for generalized linear hypothesis

C1. Technical Details for the Omnibus Test (including Proof of Theorem 2)

Recall the asymptotic variance matrix of the scaled FGLS estimator $\sqrt{n}(\widehat{\boldsymbol{\beta}} - \boldsymbol{\beta})$ can be represented by to

$$\boldsymbol{\Omega}_{\boldsymbol{\beta}} = \frac{1}{m\sigma_z^2}\left(\boldsymbol{\Sigma}_{\boldsymbol{e}} + m\boldsymbol{\Sigma}_{\boldsymbol{\phi}}\right) = \frac{1}{m\sigma_z^2}\left(\boldsymbol{A}^{\frac{1}{2}}\boldsymbol{R}\boldsymbol{A}^{\frac{1}{2}}\right),$$

where we define the diagonal matrix $\boldsymbol{A} = \text{diag}\left\{\sigma_{\phi 1}^2 + \sigma_{e1}^2, \ldots, \sigma_{\phi K}^2 + \sigma_{eK}^2\right\}$, and the implied correlation matrix is denoted by

$$\boldsymbol{R} = \begin{pmatrix} R_{11} & R_{12} & \ldots & R_{1K} \\ R_{21} & R_{22} & \ldots & R_{2K} \\ \vdots & \vdots & \ldots & \vdots \\ R_{K1} & R_{K2} & \ldots & R_{KK} \end{pmatrix}$$

with the diagonal element $R_{kk} = 1 + (m-1)\rho_0^k$, and the off-diagonal element $R_{kk'} = \rho_2^{kk'} + (m-1)\rho_1^{kk'}$. With this notation, the non-centrality parameter for omnibus test is given by

$$\tau = n\boldsymbol{\beta}^T\boldsymbol{\Omega}_{\boldsymbol{\beta}}^{-1}\boldsymbol{\beta} \propto \boldsymbol{\beta}^T\boldsymbol{A}^{-\frac{1}{2}}\boldsymbol{R}^{-1}\boldsymbol{A}^{-\frac{1}{2}}\boldsymbol{\beta} \tag{7}$$

Differentiating τ with respect to ρ_0^k and using results from matrix calculus gives

$$\frac{\partial \tau}{\partial \rho_0^k} = \boldsymbol{\beta}^T \boldsymbol{A}^{-\frac{1}{2}} \frac{\partial \boldsymbol{R}^{-1}}{\partial \rho_0^k} \boldsymbol{A}^{-\frac{1}{2}} \boldsymbol{\beta} = -\boldsymbol{\beta}^T \boldsymbol{A}^{-\frac{1}{2}} \boldsymbol{R}^{-1} \frac{\partial \boldsymbol{R}}{\partial \rho_0^k} \boldsymbol{A}^{-\frac{1}{2}} \boldsymbol{\beta} = -\boldsymbol{\beta}^T \boldsymbol{U} \frac{\partial \boldsymbol{R}}{\partial \rho_0^k} \boldsymbol{U}^T \boldsymbol{\beta},$$

where $\boldsymbol{U} = \boldsymbol{A}^{-\frac{1}{2}} \boldsymbol{R}^{-1}$. Observe that $\partial \boldsymbol{R} / \partial \rho_0^k$ is a $K \times K$ matrix with zero entries everywhere except for the (k, k)th position, which equals to a positive constant $(m-1)$. Therefore, denoting the kth column of $\boldsymbol{U}$ as $\boldsymbol{u}_{+k}$, then

$$\frac{\partial \tau}{\partial \rho_0^k} = -\boldsymbol{\beta}^T \boldsymbol{U} \frac{\partial \boldsymbol{R}}{\partial \rho_0^k} \boldsymbol{U}^T \boldsymbol{\beta} = -(m-1) \boldsymbol{\beta}^T \boldsymbol{u}_{+k} \boldsymbol{u}_{+k}^T \boldsymbol{\beta} < 0,$$

by definition of a quadratic form and $\boldsymbol{\beta} \neq \boldsymbol{0}$. This indicates the power of the omnibus test generally decreases with a larger endpoint-specific ICC. In contrast, if we differentiate τ with respect to $\rho_2^{kk'}$, we have

$$\frac{\partial \tau}{\partial \rho_2^{kk'}} = -\boldsymbol{\beta}^T \boldsymbol{U} \frac{\partial \boldsymbol{R}}{\partial \rho_2^{kk'}} \boldsymbol{U}^T \boldsymbol{\beta} = -\boldsymbol{\beta}^T \boldsymbol{u}_{+k} \boldsymbol{u}_{+k'}^T \boldsymbol{\beta},$$

which is not guaranteed to be larger or smaller than zero, and the monotonicity property is generally indeterminate.

On the other hand, some further insights can be obtained under the block exchangeable correlation structure assumption as in Li et al. (2018) and Li et al. (2020) (i.e. $\rho_0^k = \rho_0, \rho_1^{kk'} = \rho_1$ and $\rho_2^{kk'} = \rho_2$ for all k, k'). In this case, $\boldsymbol{R}$ can be written as $\boldsymbol{R} = (a-b)\boldsymbol{I}_K + b\boldsymbol{J}_K$, where $a = 1 + (m-1)\rho_0$ and $b = \rho_2 + (m-1)\rho_1$, under which case a simple inverse of the compound symmetric matrix gives

$$\boldsymbol{R}^{-1} = \frac{1}{a-b} \boldsymbol{I}_K + \frac{b}{(a-b)\{a + (K-1)b\}} \boldsymbol{J}_K.$$

Observe that $\partial \boldsymbol{R} / \partial \rho_0 = (m-1)\boldsymbol{I}_K$, and therefore

$$\frac{\partial \tau}{\partial \rho_0} = -\boldsymbol{\beta}^T \boldsymbol{U} \frac{\partial \boldsymbol{R}}{\partial \rho_0^k} \boldsymbol{U}^T \boldsymbol{\beta} = -(m-1) \boldsymbol{\beta}^T \boldsymbol{U} \boldsymbol{U}^T \boldsymbol{\beta} < 0.$$

Therefore we reach the same conclusion that the power of the omnibus test decreases with larger endpoint-specific ICC. In addition, $\partial \boldsymbol{R} / \partial \rho_2 = \boldsymbol{J}_K - \boldsymbol{I}_K$ is a $K \times K$ *symmetric hollow matrix of ones*, where all diagonal entries equal to 0 but all off-diagonal entries equal to 1.

Some further matrix algebra gives

$$\begin{aligned}
\frac{\partial \tau}{\partial \rho_2} &= -\boldsymbol{\beta}^T \boldsymbol{A}^{-\frac{1}{2}} \boldsymbol{R}^{-1} (\boldsymbol{J}_K - \boldsymbol{I}_K) \boldsymbol{R}^{-1} \boldsymbol{A}^{-\frac{1}{2}} \boldsymbol{\beta} \\
&= \boldsymbol{\beta}^T \boldsymbol{A}^{-\frac{1}{2}} \underbrace{\left[\frac{1}{(a-b)^2} \boldsymbol{I}_K - \frac{a^2 + (K-1)b^2}{(a-b)^2 \{a + (K-1)b\}^2} \boldsymbol{J}_K \right]}_{\boldsymbol{S}} \boldsymbol{A}^{-\frac{1}{2}} \boldsymbol{\beta}.
\end{aligned}$$

Notice that $\boldsymbol{S}$ is compound symmetric and has two distinct eigenvalues. The first eigenvalue is $\lambda_1 = 1/(a-b)^2 > 0$ and the second eigenvalue is

$$\lambda_2 = \frac{1}{(a-b)^2} - \frac{Ka^2 + K(K-1)b^2}{(a-b)^2 \{a + (K-1)b\}^2} = -\frac{K-1}{\{a + (K-1)b\}^2} < 0,$$

and therefore the monotonicity of τ in ρ_2 is generally indeterminate without further restrictions.

However, we further observe that the diagonal elements of $\boldsymbol{S}$ is

$$\frac{1}{(a-b)^2} - \frac{a^2 + (K-1)b^2}{(a-b)^2 \{a + (K-1)b\}^2} = (K-1)b \times \frac{(K-2)b + 2a}{(a-b)^2 \{a + (K-1)b\}^2},$$

and multiplying out $\partial \tau / \partial \rho_2$, we get

$$\begin{aligned}
\frac{\partial \tau}{\partial \rho_2} =& (K-1)b \times \frac{(K-2)b + 2a}{(a-b)^2 \{a + (K-1)b\}^2} \sum_{k=1}^{K} \widetilde{\beta}_k^2 - \frac{a^2 + (K-1)b^2}{(a-b)^2 \{a + (K-1)b\}^2} \sum_{k \neq k'} \widetilde{\beta}_k \widetilde{\beta}_{k'} \\
=& \frac{(K-1)b\{(K-2)b + 2a\} \sum_{k=1}^{K} \widetilde{\beta}_k^2 - \{a^2 + (K-1)b^2\} \sum_{k \neq k'} \widetilde{\beta}_k \widetilde{\beta}_{k'}}{(a-b)^2 \{a + (K-1)b\}^2} \qquad (8)
\end{aligned}$$

where $\widetilde{\beta}_k = \beta_k / \sigma_{yk}$ is the standardized effect size. If the K standardized effect sizes are identical such that $\widetilde{\beta}_1 = \ldots = \widetilde{\beta}_K$, then

$$\frac{\partial \tau}{\partial \rho_2} \propto \frac{(K-1)b\{(K-2)b + 2a\} - \{a^2 + (K-1)b^2 K(K-1)\}}{(a-b)^2 \{a + (K-1)b\}^2} = \frac{-K(K-1)}{\{a + (K-1)b\}^2} < 0.$$

Therefore, the power of omnibus test is decreasing with a larger intra-subject ICC ρ_2. The proof on monotonicty in the inter-subject between-endpoint ICC ρ_1 can be shown in an exact same fashion because ρ_1 enters $\boldsymbol{R}$ through the same off-diagonal element as ρ_2 but with a positive multiplier $(m-1)$.

C2. Technical Details for the Test for Treatment Effect Homogeneity

Similar to the Omnibus test, the relationship between $\rho_1^{kk'}$, $\rho_2^{kk'}$ $\forall\ k \neq k'$ and the power of the test for treatment effect homogeneity is generally indeterminate. However, under the parsimonious block exchangeable correlation structure such that $\rho_0^k = \rho_0$, $\rho_1^{kk'} = \rho_1$, $\rho_2^{kk'} = \rho_2\ \forall\ k, k'$ and assuming equal marginal variances such that $\sigma_{yk}^2 = \sigma_{yk'}^2\ \forall\ k \neq k'$, we can obtain the following Theorem.

Theorem S2 *(Test for treatment effect homogeneity) With all other design parameters fixed, a larger value of the endpoint-specific ICC, $\rho_0^k\ \forall\ k$, is always associated with a smaller power of the test for treatment effect homogeneity (a larger sample size), while the relationship between $\rho_1^{kk'}$, $\rho_2^{kk'}\ \forall\ k \neq k'$ and the power of the test for treatment effect homogeneity is generally indeterminate. However, under the parsimonious block exchangeable correlation structure such that $\rho_0^k = \rho_0$, $\rho_1^{kk'} = \rho_1$, $\rho_2^{kk'} = \rho_2\ \forall\ k, k'$ and assuming equal marginal variances such that $\sigma_{yk}^2 = \sigma_{yk'}^2\ \forall\ k \neq k'$, a larger value of the endpoint-specific ICC, ρ_0, is always associated with a smaller power of the test for homogeneity (a larger sample size), whereas larger values of between-endpoint ICCs, ρ_1 or ρ_2, are always associated with a larger power of the test for homogeneity (smaller required sample size).*

Below, we present the technical details to support the above results. Recall that the test for treatment effect homogeneity corresponds to a contrast matrix $\boldsymbol{L} = (\boldsymbol{e}_1 - \boldsymbol{e}_2, \boldsymbol{e}_2 - \boldsymbol{e}_3, \ldots, \boldsymbol{e}_{K-1} - \boldsymbol{e}_K)^T$, and $\boldsymbol{L\beta} = (\beta_1 - \beta_2, \ldots, \beta_{K-1} - \beta_K)^T = (\delta_{12}, \ldots, \delta_{K-1,K})^T = \boldsymbol{B}$. Therefore, the non-centrality parameter is given by

$$\tau = n(\boldsymbol{L\beta})^T(\boldsymbol{L\Omega_\beta L}^T)^{-1}(\boldsymbol{L\beta}) \propto (\boldsymbol{L\beta})^T(\boldsymbol{LA}^{\frac{1}{2}}\boldsymbol{RA}^{\frac{1}{2}}\boldsymbol{L}^T)^{-1}(\boldsymbol{L\beta}) = \boldsymbol{B}^T\boldsymbol{M}^{-1}\boldsymbol{B},$$

where the (j,j)th diagonal entry of $\boldsymbol{M}$ equals to $\omega_{jj} + \omega_{j+1,j+1}$, and the (j,l)th off-diagonal entry of $\boldsymbol{M}$ equal to $\omega_{jl} + \omega_{j+1,l+1} - \omega_{j,l+1} - \omega_{j+1,l}$. Note that $\omega_{kk'}$ is defined in equation (6) of the main paper, and for convenience, we rewrite $\omega_{kk} = \omega_k^2$, which is defined in the equation (5) of the main paper. Differentiating τ with respect to ρ_0^k gives

$$\frac{\partial\tau}{\partial\rho_0^k} = -\boldsymbol{B}^T\boldsymbol{M}^{-1}\frac{\partial\boldsymbol{M}}{\partial\rho_0^k}\boldsymbol{M}^{-1}\boldsymbol{B} = -\boldsymbol{B}^T\boldsymbol{U}\frac{\partial\boldsymbol{M}}{\partial\rho_0^k}\boldsymbol{U}^T\boldsymbol{B},$$

where with a slight abuse of notation we define $\boldsymbol{U} = \boldsymbol{M}^{-1} = \boldsymbol{U}^T$. Above all, we observe

that $\partial \boldsymbol{M}/\partial \rho_0^k$ is a $(K-1)\times(K-1)$ matrix. When $k \in \{1, K\}$, this matrix has an element

$$\left[\frac{\partial \boldsymbol{M}}{\partial \rho_0^k}\right]_{kk} = (m-1)\sigma_{yk}^2,$$

but zero everywhere else. When $2 \leq k \leq K-1$, then this matrix has elements

$$\begin{aligned}\left[\frac{\partial \boldsymbol{M}}{\partial \rho_0^k}\right]_{kk} &= \left[\frac{\partial \boldsymbol{M}}{\partial \rho_0^k}\right]_{k-1,k-1} &= (m-1)\sigma_{yk}^2 \\ \left[\frac{\partial \boldsymbol{M}}{\partial \rho_0^k}\right]_{k-1,k} &= \left[\frac{\partial \boldsymbol{M}}{\partial \rho_0^k}\right]_{k,k-1} &= -(m-1)\sigma_{yk}^2,\end{aligned}$$

but zero everywhere else. If we define the kth column of $\boldsymbol{U}$ as $\boldsymbol{u}_{+k}$, we can multiply out $\partial\tau/\partial\rho_0^k$ and get

$$\begin{aligned}\frac{\partial \tau}{\partial \rho_0^k} &= -\boldsymbol{B}^T\boldsymbol{U}\frac{\partial \boldsymbol{M}}{\partial \rho_0^k}\boldsymbol{U}^T\boldsymbol{B} \\ &= \begin{cases} -(m-1)\sigma_{yk}^2\boldsymbol{B}^T\boldsymbol{u}_{+k}\boldsymbol{u}_{+k}^T\boldsymbol{B} < 0, & \text{when } k = 1, K \\ -(m-1)\boldsymbol{B}^T(\boldsymbol{u}_{+,k-1} - \boldsymbol{u}_{+k})(\boldsymbol{u}_{+,k-1} - \boldsymbol{u}_{+k})^T\boldsymbol{B} < 0, & \text{when } 2 \leq k \leq K-1, \end{cases}\end{aligned}$$

which implies the power of test for treatment effect homogeneity decreases with a larger endpoint-specific ICC ρ_0^k (since the effect size $\boldsymbol{B} \neq \boldsymbol{0}$ under the alternative). However, for the other two types of ICC parameters, it turns out that there are too many patterns for the derivatives of $\boldsymbol{M}$, and the final derivative is not always guaranteed to be positive or negative, and thus we cannot determine monotonicity without further assumptions.

Next assuming the implied correlation structure for each cluster is block exchangeable such that $\rho_0^k = \rho_0, \rho_1^{kk'} = \rho_1$ and $\rho_2^{kk'} = \rho_2$ for all k, k', we also differentiate τ with respect to ρ_0 and obtain

$$\frac{\partial \tau}{\partial \rho_0} = -\boldsymbol{B}^T\boldsymbol{M}^{-1}\frac{\partial \boldsymbol{M}}{\partial \rho_0^k}\boldsymbol{M}^{-1}\boldsymbol{B} = -\boldsymbol{B}^T\boldsymbol{U}\frac{\partial \boldsymbol{M}}{\partial \rho_0}\boldsymbol{U}^T\boldsymbol{B},$$

where $\boldsymbol{U} = \boldsymbol{M}^{-1} = \boldsymbol{U}^T$, but $\boldsymbol{M}$ is now a matrix with diagonal element $\sigma_{yk}^2\{1+(m-1)\rho_0\}$ and off-diagonal element as $\sigma_{yk}\sigma_{yk'}\{\rho_2 + (m-1)\rho_1\}$. Again defining the kth column of $\boldsymbol{U}$

as $\boldsymbol{u}_{+k}$, we can write

$$\begin{aligned}
\frac{\partial \tau}{\partial \rho_0} =& -(m-1)\boldsymbol{B}^T \left[\left\{\sigma_{y1}^2 \boldsymbol{u}_{+1}\boldsymbol{u}_{+1}^T + \sigma_{y2}^2(\boldsymbol{u}_{+1} - \boldsymbol{u}_{+2})\boldsymbol{u}_{+1}^T\right\}\right.\\
&+ \left\{-\sigma_{y2}^2(\boldsymbol{u}_{+1} - \boldsymbol{u}_{+2})\boldsymbol{u}_{+2}^T + \sigma_{y3}^2(\boldsymbol{u}_{+2} - \boldsymbol{u}_{+3})\boldsymbol{u}_{+2}^T\right\}\\
&+ \left\{-\sigma_{y3}^2(\boldsymbol{u}_{+2} - \boldsymbol{u}_{+3})\boldsymbol{u}_{+3}^T + \sigma_{y4}^2(\boldsymbol{u}_{+3} - \boldsymbol{u}_{+4})\boldsymbol{u}_{+3}^T\right\} + \dots\\
&+ \left.\left\{-\sigma_{y,K-1}^2(\boldsymbol{u}_{+,K-2} - \boldsymbol{u}_{+,K-1})\boldsymbol{u}_{+,K-1}^T + \sigma_{yK}^2 \boldsymbol{u}_{+,K-1}\boldsymbol{u}_{+,K-1}^T\right\}\right] \boldsymbol{B}\\
=& -(m-1)\boldsymbol{B}^T \left[\sigma_{y1}^2 \boldsymbol{u}_{+1}\boldsymbol{u}_{+1}^T + \sum_{k=2}^{K-1} \sigma_{yk}^2 (\boldsymbol{u}_{+,k-1} - \boldsymbol{u}_{+k})(\boldsymbol{u}_{+,k-1} - \boldsymbol{u}_{+k})^T + \sigma_{yK}^2 \boldsymbol{u}_{+,K-1}\boldsymbol{u}_{+,K-1}^T\right] \boldsymbol{B}\\
<& 0,
\end{aligned}$$

because the kernel of the quadratic form is a sequential sum of squares. Therefore, the power of the omnibus test also decreases with a larger (common) endpoint-specific ICC ρ_0.

Next, we differentiate τ with respect to ρ_2.

$$\frac{\partial \tau}{\partial \rho_2} = -\boldsymbol{B}^T \boldsymbol{U} \frac{\partial \boldsymbol{M}}{\partial \rho_2} \boldsymbol{U}^T \boldsymbol{B}.$$

Define $a_{kk'} = \sigma_{yk}\sigma_{yk'}$, and define $\boldsymbol{O}$ a $(K-1) \times (K-1)$ matrix whose (j,l)th element is $a_{jl} + a_{j+1,l+1} - a_{j+1,l} - a_{j,l+1}$. Then we have the $(K-1) \times (K-1)$ symmetric matrix

$$\frac{\partial \boldsymbol{M}}{\partial \rho_2} = \boldsymbol{O} - \begin{bmatrix} a_{11} + a_{22} & -a_{22} & 0 & \dots & 0 \\ -a_{22} & a_{22} + a_{33} & -a_{33} & \dots & 0 \\ 0 & -a_{33} & a_{33} + a_{44} & \dots & 0 \\ \vdots & \vdots & \vdots & \vdots & \vdots \\ 0 & 0 & 0 & \dots & a_{K-1,K-1} + a_{KK} \end{bmatrix}$$

This allows us to further obtain

$$
\begin{aligned}
\boldsymbol{U}\frac{\partial \boldsymbol{M}}{\partial \rho_2}\boldsymbol{U}^T =& \sum_{j=1}^{K-1}\left\{\sum_{k=1}^{K-1}\boldsymbol{u}_{+k}\boldsymbol{u}_{+j}^T(a_{jk}+a_{j+1,k+1}-a_{j+1,k}-a_{j,k+1})\right\} - \{\boldsymbol{u}_{+1}\boldsymbol{u}_{+1}^T(a_{11}+a_{22})-\boldsymbol{u}_{+2}\boldsymbol{u}_{+1}^T a_{22}\} \\
&- \{-\boldsymbol{u}_{+,K-2}\boldsymbol{u}_{+,K-1}^T a_{K-1,K-1}+\boldsymbol{u}_{+,K-1}\boldsymbol{u}_{+,K-1}^T(a_{K-1,K-1}+a_{KK})\} \\
&- \sum_{j=2}^{K-2}\{-\boldsymbol{u}_{+,j-1}\boldsymbol{u}_{+j}^T a_{jj}+\boldsymbol{u}_{+,j}\boldsymbol{u}_{+j}^T(a_{jj}+a_{j+1,j+1})-\boldsymbol{u}_{+,j+1}\boldsymbol{u}_{+j}^T a_{j+1,j+1}\} \\
=& \sum_{j=1}^{K-1}\left\{\sum_{k=1}^{K-1}\boldsymbol{u}_{+k}\boldsymbol{u}_{+j}^T(a_{jk}+a_{j+1,k+1}-a_{j+1,k}-a_{j,k+1})+\boldsymbol{u}_{+,j-1}\boldsymbol{u}_{+j}^T a_{jj}\right. \\
&\left. -\boldsymbol{u}_{+j}\boldsymbol{u}_{+j}^T(a_{jj}+a_{j+1,j+1})+\boldsymbol{u}_{+,j+1}\boldsymbol{u}_{+j}^T a_{j+1,j+1}\right\},
\end{aligned}
$$

where in tha last equation we have defined $\boldsymbol{u}_{+0}=\boldsymbol{u}_{+K}=\boldsymbol{0}$ for convenience. It is easy to see that the last expression is not guaranteed to be positive or negative and therefore the monotonicty relationship is indeterminate without further assumption. However, under an additional assumption that the marginal variance are equal, we have $\partial \boldsymbol{M}/\partial\rho_2$ as a tri-diagonal matrix given by

$$
\frac{\partial \boldsymbol{M}}{\partial \rho_2} \propto \begin{bmatrix} -2 & 1 & 0 & \dots & 0 \\ 1 & -2 & 1 & \dots & 0 \\ 0 & 1 & -2 & \dots & 0 \\ \vdots & \vdots & \vdots & \vdots & \vdots \\ 0 & 0 & 0 & 1 & -2 \end{bmatrix}
$$

and

$$
\begin{aligned}
\boldsymbol{U}\frac{\partial \boldsymbol{M}}{\partial \rho_2}\boldsymbol{U}^T \propto& (-2\boldsymbol{u}_{+1}+\boldsymbol{u}_{+2})\boldsymbol{u}_{+1}^T+(\boldsymbol{u}_{+1}-2\boldsymbol{u}_{+2}+\boldsymbol{u}_{+3})\boldsymbol{u}_{+2}^T+\ldots+(\boldsymbol{u}_{+,k-1}-2\boldsymbol{u}_{+k}+\boldsymbol{u}_{+,k+1})\boldsymbol{u}_{+k}^T \\
&+\ldots+(\boldsymbol{u}_{+,K-2}-2\boldsymbol{u}_{+,K-1})\boldsymbol{u}_{+,k-1}^T \\
=& -\boldsymbol{u}_{+1}\boldsymbol{u}_{+1}^T-(\boldsymbol{u}_{+1}-\boldsymbol{u}_{+2})(\boldsymbol{u}_{+1}-\boldsymbol{u}_{+2})^T-\ldots-\boldsymbol{u}_{+,K-1}\boldsymbol{u}_{+,K-1}^T<0
\end{aligned}
$$

That is

$$
\frac{\partial \tau}{\partial \rho_2} = -\boldsymbol{B}^T\boldsymbol{U}\frac{\partial \boldsymbol{M}}{\partial \rho_2}\boldsymbol{U}^T\boldsymbol{B} > 0,
$$

suggesting the power of the test of treatment effect homogeneity is an increasing function

of ρ_2. The monotonicity result for ρ_1 follows the exact same step as above for ρ_2, up to scalar constant $(m-1)$.

Web Appendix D: Technical Details for the Intersection-Union Test

The proof for Theorem 3 is similar to that in Li et al. (2020), which uses the following intermediate result (Theorem 4.3.6 of Tong (2014)). This intermediate result is re-stated as follows.

Let $\boldsymbol{\Sigma}$ denote a $L \times L$ positive definite matrix and assume $\boldsymbol{X}$ to have a density function $f(\boldsymbol{x})$ of the form:

$$f(\boldsymbol{x}) = |\boldsymbol{\Sigma}|^{-1/2} g(\boldsymbol{x}'\boldsymbol{\Sigma}^{-1}\boldsymbol{x}), \tag{9}$$

where the function $g(\cdot)$ satisfies

$$\int_0^\infty r^{L-1} g(r^2) dr < \infty. \tag{10}$$

Let $\boldsymbol{P} = (p_{ij})$, $\boldsymbol{T} = (t_{ij})$ be two $L \times L$ positive definite matrices. If $p_{ij} \geq t_{ij}$ holds for all i, j, then

$$P_{\boldsymbol{\Sigma}=\boldsymbol{P}}[\cap_{i=1}^{L}\{X_i \leq a_i\}] \geq P_{\boldsymbol{\Sigma}=\boldsymbol{T}}[\cap_{i=1}^{L}\{X_i \leq a_i\}] \tag{11}$$

holds for every $\boldsymbol{a} = (a_1, \ldots, a_L)'$. Furthermore, the inequality is strict if $p_{ij} > t_{ij}$ holds for some i, j and if the support of f is unbounded.

We give the proof for ρ_0^k, and the proofs for $\rho_1^{kk'}, \rho_2^{kk'}$ can be shown in a similar argument. First note, the multivariate normal and multivariate t-distribution are of the form (9). Recall the vector of test statistics $\boldsymbol{\zeta}$ asymptotically follows a multivariate normal distribution with mean $\boldsymbol{\eta} = (\sqrt{n}\beta_1/\omega_1, \ldots, \sqrt{n}\beta_K/\omega_K)^T$ and covariance matrix $\boldsymbol{\Phi}$ (this is also the correlation matrix since the marginal variances are all one), whose the diagonal and off-diagonal elements are given by

$$\phi_{kk'} = \mathbb{1}\{k = k'\} + \frac{\omega_{kk'}}{\omega_k \omega_{k'}} \mathbb{1}\{k \neq k'\}. \tag{12}$$

Recall Equation (5) in the main text implies that σ_k^2 is an increasing function of ρ_0^k, and (12) above implies that $\phi_{kk'} = \sigma_{kk'}/(\sigma_k\sigma_{k'})$ is a decreasing function of ρ_0^k. In addition, the kth element of $\boldsymbol{\eta}$, $\eta_k = \sqrt{n}\beta_k/\sigma_k$, is a decreasing function of ρ_0^k. Then for two values $\kappa_1 < \kappa_2$ for ρ_0^k, let $\boldsymbol{P} = p_{kk'}$ be the positive definite covariance matrix in (12) with $\rho_0^k = \kappa_1$, and $\boldsymbol{T} = t_{kk'}$ be the positive definite covariance matrix with $\rho_0^k = \kappa_2$. When all other parameters are fixed, $\kappa_1 < \kappa_2$ implies $p_{ij} > t_{ij}$ for all $k \neq k'$, and $p_{ij} = t_{ij}$ for all $k = k'$. It also implies that $\eta_k^{\kappa_1} > \eta_k^{\kappa_2}$ for the mean values associated with κ_1 and κ_2. Equation (11) also implies

$$P_{\boldsymbol{\Sigma}=\boldsymbol{P}}[\cap_{i=1}^{L}\{X_i \geq a_i\}] \geq P_{\boldsymbol{\Sigma}=\boldsymbol{T}}[\cap_{i=1}^{L}\{X_i \geq a_i\}] \tag{13}$$

Therefore, let $\boldsymbol{Z} = (z_1, \ldots, z_k)^T$ have a distribution with mean zeros and correlation matrix P,

$$\begin{aligned}
\text{power}(\boldsymbol{\Phi} = \boldsymbol{P}) = \text{Prob}_{\boldsymbol{\Phi}=\boldsymbol{P}}\left(\bigcap_{k=1}^{K}\{w_k > c\}\right) &= \text{Prob}_{\boldsymbol{\Phi}=\boldsymbol{P}}\left(\bigcap_{k=1}^{K}\{z_k > c - \eta_k^{\kappa_1}\}\right) \\
&> \text{Prob}_{\boldsymbol{\Phi}=\boldsymbol{T}}\left(\bigcap_{k=1}^{K}\{z_k > c - \eta_k^{\kappa_1}\}\right) \text{ by (13)} \\
&> \text{Prob}_{\boldsymbol{\Phi}=\boldsymbol{T}}\left(\bigcap_{k=1}^{K}\{z_k > c - \eta_k^{\kappa_2}\}\right) \\
&= \text{power}(\boldsymbol{\Phi} = \boldsymbol{T})
\end{aligned}$$

Web Appendix E: Derivation of the Variance Expression of Treatment Effect Estimators under Variable Cluster Sizes

Under variable cluster sizes, When $m_i \neq m_{i'}$, we have $\boldsymbol{W}_i = \mathbf{1}_{m_i} \otimes (\boldsymbol{I}_K, \boldsymbol{I}_K(z_i - \overline{z}))$, and similar to results in Section 3.1 of the main paper,

$$\boldsymbol{V}_i^{-1} = \boldsymbol{I}_{m_i} \otimes \boldsymbol{\Sigma}_{\boldsymbol{e}}^{-1} + \boldsymbol{J}_{m_i} \otimes \frac{1}{m_i}\left\{(\boldsymbol{\Sigma}_{\boldsymbol{e}} + m_i\boldsymbol{\Sigma}_{\boldsymbol{\phi}})^{-1} - \boldsymbol{\Sigma}_{\boldsymbol{e}}^{-1}\right\}.$$

Then we can write

$$
\begin{aligned}
\boldsymbol{U_n} = \sum_{i=1}^{n} \boldsymbol{W_i}^T \boldsymbol{V_i}^{-1} \boldsymbol{W_i} = & \sum_{i=1}^{n} m_i \begin{pmatrix} 1 & (z_i - \overline{z}) \\ (z_i - \overline{z}) & (z_i - \overline{z})^2 \end{pmatrix} \otimes \boldsymbol{\Sigma_e}^{-1} + \\
& \sum_{i=1}^{n} m_i \begin{pmatrix} 1 & (z_i - \overline{z}) \\ (z_i - \overline{z}) & (z_i - \overline{z})^2 \end{pmatrix} \otimes \left\{ (\boldsymbol{\Sigma_e} + m_i \boldsymbol{\Sigma_\phi})^{-1} - \boldsymbol{\Sigma_e}^{-1} \right\} \\
= & \sum_{i=1}^{n} m_i \begin{pmatrix} 1 & (z_i - \overline{z}) \\ (z_i - \overline{z}) & (z_i - \overline{z})^2 \end{pmatrix} \otimes (\boldsymbol{\Sigma_e} + m_i \boldsymbol{\Sigma_\phi})^{-1} .
\end{aligned}
$$

It follows that the limit of the variance of the scaled FGLS estimator $\sqrt{n}(\widehat{\boldsymbol{\beta}} - \boldsymbol{\beta})$, takes the form

$$
\boldsymbol{\Omega_\beta} = \lim_{n \to \infty} \left[\frac{1}{n} \sum_{i=1}^{n} (z_i - \overline{z})^2 m_i \left(\boldsymbol{\Sigma_e} + m_i \boldsymbol{\Sigma_\phi} \right)^{-1} \right]^{-1} = \frac{1}{\sigma_z^2} \boldsymbol{\Sigma_\phi} \left[\mathbb{E} \left\{ m_i \left(m_i \boldsymbol{I}_K + \boldsymbol{A} \right)^{-1} \right\} \right]^{-1},
$$

where we define $\boldsymbol{A} = (\boldsymbol{\Sigma_e} + \boldsymbol{\Sigma_\phi}) \boldsymbol{\Sigma_\phi}^{-1} - \boldsymbol{I}_K = \boldsymbol{\Sigma_e} \boldsymbol{\Sigma_\phi}^{-1}$. To proceed, we define $d = m_i - \overline{m}$ as the difference between the ith cluster size and the mean cluster size, then

$$
\begin{aligned}
\mathbb{E}\{m_i (m_i \boldsymbol{I}_K + \boldsymbol{A})\}^{-1} = & \mathbb{E} \left\{ (\overline{m} + d) \left((\overline{m} + d) \boldsymbol{I}_K + \boldsymbol{A} \right)^{-1} \right\} \\
= & \mathbb{E} \left\{ (\overline{m} + d) (\overline{m} \boldsymbol{I}_K + \boldsymbol{A})^{-1} \left(\boldsymbol{I}_K + d (\overline{m} \boldsymbol{I}_K + \boldsymbol{A})^{-1} \right)^{-1} \right\} . \qquad (14)
\end{aligned}
$$

By the Neumann series for matrix inverse (Beilina et al., 2017), we have

$$
\left(\boldsymbol{I}_K + d (\overline{m} \boldsymbol{I}_K + \boldsymbol{A})^{-1} \right)^{-1} = \sum_{q=0}^{\infty} (-1)^q d^q \left(\overline{m} \boldsymbol{I}_K + \boldsymbol{A} \right)^{-q} , \qquad (15)
$$

for $-\|(\overline{m} \boldsymbol{I}_K + \boldsymbol{A})^{-1}\|^{-1} < d < \|(\overline{m} \boldsymbol{I}_K + \boldsymbol{A})^{-1}\|^{-1}$ to ensure that the series converge. Since d measures the deviation from the mean cluster size and the two bounds increase as a function of the mean cluster sizes, the convergence is likely to hold for moderate to large $\overline{m}$. For the second-order approximation, we discard all terms of d^q with $q > 2$ and then approximate

$$
\begin{aligned}
& \mathbb{E}\{m_i (m_i \boldsymbol{I}_K + \boldsymbol{A})\}^{-1} \\
\approx & \mathbb{E}(\overline{m} + d)(\overline{m} \boldsymbol{I}_K + \boldsymbol{A})^{-1} - \mathbb{E} \left\{ d (\overline{m} + d) \right\} (\overline{m} \boldsymbol{I}_K + \boldsymbol{A})^{-2} + \mathbb{E}(d^2 \overline{m}) (\overline{m} \boldsymbol{I}_K + \boldsymbol{A})^{-3}
\end{aligned}
$$

By definition, $\mathbb{E}(d) = 0$ and $\mathbb{E}(d^2) = \text{CV}^2\overline{m}^2$, we can write

$$
\begin{aligned}
&\mathbb{E}\{m_i(m_i\boldsymbol{I}_K + \boldsymbol{A})\}^{-1} \approx \overline{m}(\overline{m}\boldsymbol{I}_K + \boldsymbol{A})^{-1}\left[\boldsymbol{I}_K - \text{CV}^2\left\{\overline{m}(\overline{m}\boldsymbol{I}_K + \boldsymbol{A})^{-1} - \overline{m}^2(\overline{m}\boldsymbol{I}_K + \boldsymbol{A})^{-2}\right\}\right] \\
=&\overline{m}(\overline{m}\boldsymbol{I}_K + \boldsymbol{A})^{-1}\left[\boldsymbol{I}_K - \text{CV}^2\left\{\overline{m}(\overline{m}\boldsymbol{I}_K + \boldsymbol{A})^{-1}(\boldsymbol{I}_K - \overline{m}(\overline{m}\boldsymbol{I}_K + \boldsymbol{A})^{-1})\right\}\right] \\
=&\overline{m}(\overline{m}\boldsymbol{I}_K + \boldsymbol{A})^{-1}\left[\boldsymbol{I}_K - \text{CV}^2\left\{\overline{m}(\overline{m}\boldsymbol{I}_K + \boldsymbol{A})^{-1}\boldsymbol{A}(\overline{m}\boldsymbol{I}_K + \boldsymbol{A})^{-1}\right\}\right].
\end{aligned}
$$

Plugging in the expression of $\boldsymbol{A}$, we have

$$
\boldsymbol{\Omega}_{\boldsymbol{\beta}} \approx \frac{(\boldsymbol{\Sigma}_{\boldsymbol{e}} + \overline{m}\boldsymbol{\Sigma}_{\boldsymbol{\phi}})}{\overline{m}\sigma_z^2} \times \left[\boldsymbol{I}_K - \text{CV}^2\left\{\overline{m}\boldsymbol{\Sigma}_{\boldsymbol{\phi}}(\boldsymbol{\Sigma}_{\boldsymbol{e}} + \overline{m}\boldsymbol{\Sigma}_{\boldsymbol{\phi}})^{-1}\boldsymbol{\Sigma}_{\boldsymbol{e}}(\boldsymbol{\Sigma}_{\boldsymbol{e}} + \overline{m}\boldsymbol{\Sigma}_{\boldsymbol{\phi}})^{-1}\right\}\right]^{-1}.
$$

Notice that we can approximate the variance expression up to any finite order which requires higher moments of the distribution of cluster sizes. For example, the fourth-order approximation discards all terms of d^q with $q > 4$ and then approximate

$$
\begin{aligned}
&\mathbb{E}\{m_i(m_i\boldsymbol{I}_K + \boldsymbol{A})\}^{-1} \approx \overline{m}(\overline{m}\boldsymbol{I}_K + \boldsymbol{A})^{-1}\left[\boldsymbol{I}_K - \text{CV}^2\left\{\overline{m}(\overline{m}\boldsymbol{I}_K + \boldsymbol{A})^{-1}\boldsymbol{A}(\overline{m}\boldsymbol{I}_K + \boldsymbol{A})^{-1}\right\}\right] + \\
&(\overline{m}\boldsymbol{I}_K + \boldsymbol{A})^{-1}\left\{\mathbb{E}(d^3)(\overline{m}\boldsymbol{I}_K + \boldsymbol{A})^{-2} - \mathbb{E}(d^4)(\overline{m}\boldsymbol{I}_K + \boldsymbol{A})^{-3}\right\}\left\{\boldsymbol{I}_K - \overline{m}(\overline{m}\boldsymbol{I}_K + \boldsymbol{A})^{-1}\right\}
\end{aligned}
$$

Recall that the skewness is the third central moment of a random variable divided by the standard deviation to the third, and kurtosis is the fourth central moment of a random variable divided by the squared variance minus 3, then we have

$$
\mathbb{E}(d^3) = \text{CV}^3\overline{m}^3 \times \text{skew}, \qquad \mathbb{E}(d^4) = \text{CV}^4\overline{m}^4 \times (\text{kurt} + 3),
$$

in which case we obtain

$$
\begin{aligned}
&\mathbb{E}\{m_i(m_i\boldsymbol{I}_K + \boldsymbol{A})\}^{-1} \\
\approx&\overline{m}(\overline{m}\boldsymbol{I}_K + \boldsymbol{A})^{-1}\left[\boldsymbol{I}_K - \text{CV}^2\left\{\overline{m}(\overline{m}\boldsymbol{I}_K + \boldsymbol{A})^{-1}\boldsymbol{A}(\overline{m}\boldsymbol{I}_K + \boldsymbol{A})^{-1}\right\}\right] + \\
&\overline{m}(\overline{m}\boldsymbol{I}_K + \boldsymbol{A})^{-1}\left\{\text{CV}^3\overline{m}^2(\overline{m}\boldsymbol{I}_K + \boldsymbol{A})^{-2}\text{skew} - \text{CV}^4\overline{m}^3(\overline{m}\boldsymbol{I}_K + \boldsymbol{A})^{-3}(\text{kurt} + 3)\right\}\boldsymbol{A}(\overline{m}\boldsymbol{I}_K + \boldsymbol{A})^{-1} \\
=&\overline{m}(\overline{m}\boldsymbol{I}_K + \boldsymbol{A})^{-1}\left[\boldsymbol{I}_K - \xi_f \times \text{CV}^2\left\{\overline{m}(\overline{m}\boldsymbol{I}_K + \boldsymbol{A})^{-1}\boldsymbol{A}(\overline{m}\boldsymbol{I}_K + \boldsymbol{A})^{-1}\right\}\right],
\end{aligned}
$$

where the additional factor incorporating the skewness and kurtosis of $f(m_i)$ is given by

$$
\xi_f = 1 - \text{CV}\overline{m}(\overline{m}\boldsymbol{I}_K + \boldsymbol{A})^{-1}\text{skew} + \text{CV}^2\overline{m}^2(\overline{m}\boldsymbol{I}_K + \boldsymbol{A})^{-2}(\text{kurt} + 3).
$$

Plugging in the expression of $\boldsymbol{A}$, we get the fourth-order approximation as

$$\boldsymbol{\Omega}_{\boldsymbol{\beta}} \approx \frac{(\boldsymbol{\Sigma}_{\boldsymbol{e}} + \overline{m}\boldsymbol{\Sigma}_{\boldsymbol{\phi}})}{\overline{m}\sigma_z^2} \times \left[\boldsymbol{I}_K - \xi_f \times \mathrm{CV}^2 \left\{\overline{m}\boldsymbol{\Sigma}_{\boldsymbol{\phi}}(\boldsymbol{\Sigma}_{\boldsymbol{e}} + \overline{m}\boldsymbol{\Sigma}_{\boldsymbol{\phi}})^{-1}\boldsymbol{\Sigma}_{\boldsymbol{e}}(\boldsymbol{\Sigma}_{\boldsymbol{e}} + \overline{m}\boldsymbol{\Sigma}_{\boldsymbol{\phi}})^{-1}\right\}\right]^{-1},$$

where

$$\xi_f = 1 - \mathrm{CV}\overline{m}\boldsymbol{\Sigma}_{\boldsymbol{\phi}}(\boldsymbol{\Sigma}_{\boldsymbol{e}} + \overline{m}\boldsymbol{\Sigma}_{\boldsymbol{\phi}})^{-1}\mathrm{skew} + \mathrm{CV}^2\overline{m}^2\boldsymbol{\Sigma}_{\boldsymbol{\phi}}(\boldsymbol{\Sigma}_{\boldsymbol{e}} + \overline{m}\boldsymbol{\Sigma}_{\boldsymbol{\phi}})^{-1}\boldsymbol{\Sigma}_{\boldsymbol{\phi}}(\boldsymbol{\Sigma}_{\boldsymbol{e}} + \overline{m}\boldsymbol{\Sigma}_{\boldsymbol{\phi}})^{-1}(\mathrm{kurt}+3).$$

Web Appendix F: Additional Results for Data Application

In Section 5 of the main paper, if the investigators are interested in obtaining the power based on the test for treatment effect homogeneity, solving equation (8) with variance (11) in the paper suggests that $n = 38$ clusters are required to detect effect sizes $(\beta_1, \beta_2) = (0.35, 0.7) \times \boldsymbol{\sigma}_y$ with 80% power and 5% significance level. Under a similar setting as described in the paper, Web Figure 6 presents a power contour for the test for treatment effect homogeneity when the inter-subject between-endpoint ICC $\rho_1^{12}/\rho_0^1 \in [0.1, 1.5]$, intra-subject ICC $\rho_2^{12} \in \{0.4, 0.79\}$ for the effect size $(\beta_1, \beta_2) = 0.3 \times \boldsymbol{\sigma}_y$. The predicted power ranges between $[0.25, 0.98]$, and it is evident that power decreases with larger values of ρ_0^1 and smaller values of ρ_2^{12}.

Web Appendix G: Web Figures

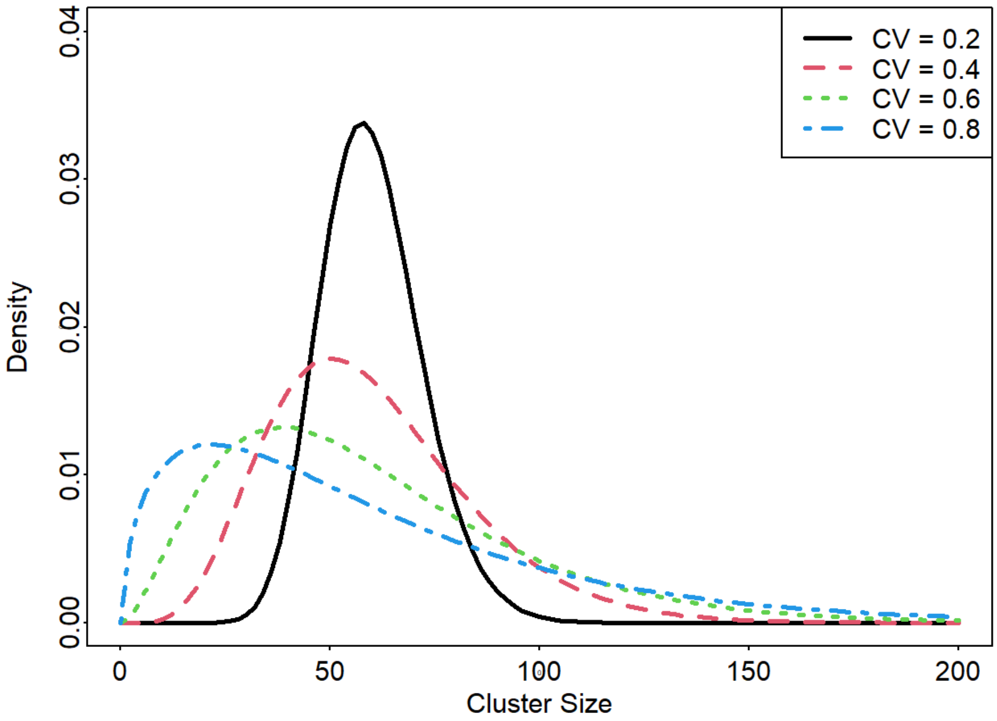

Web Figure 1: Density plot of cluster sizes under various coefficient of variations when the mean cluster size $\overline{m} = 60$, assuming the cluster size follows a Gamma distribution with shape and scale parameter $1/\mathrm{CV}^2$ and $\overline{m}\mathrm{CV}^2$.

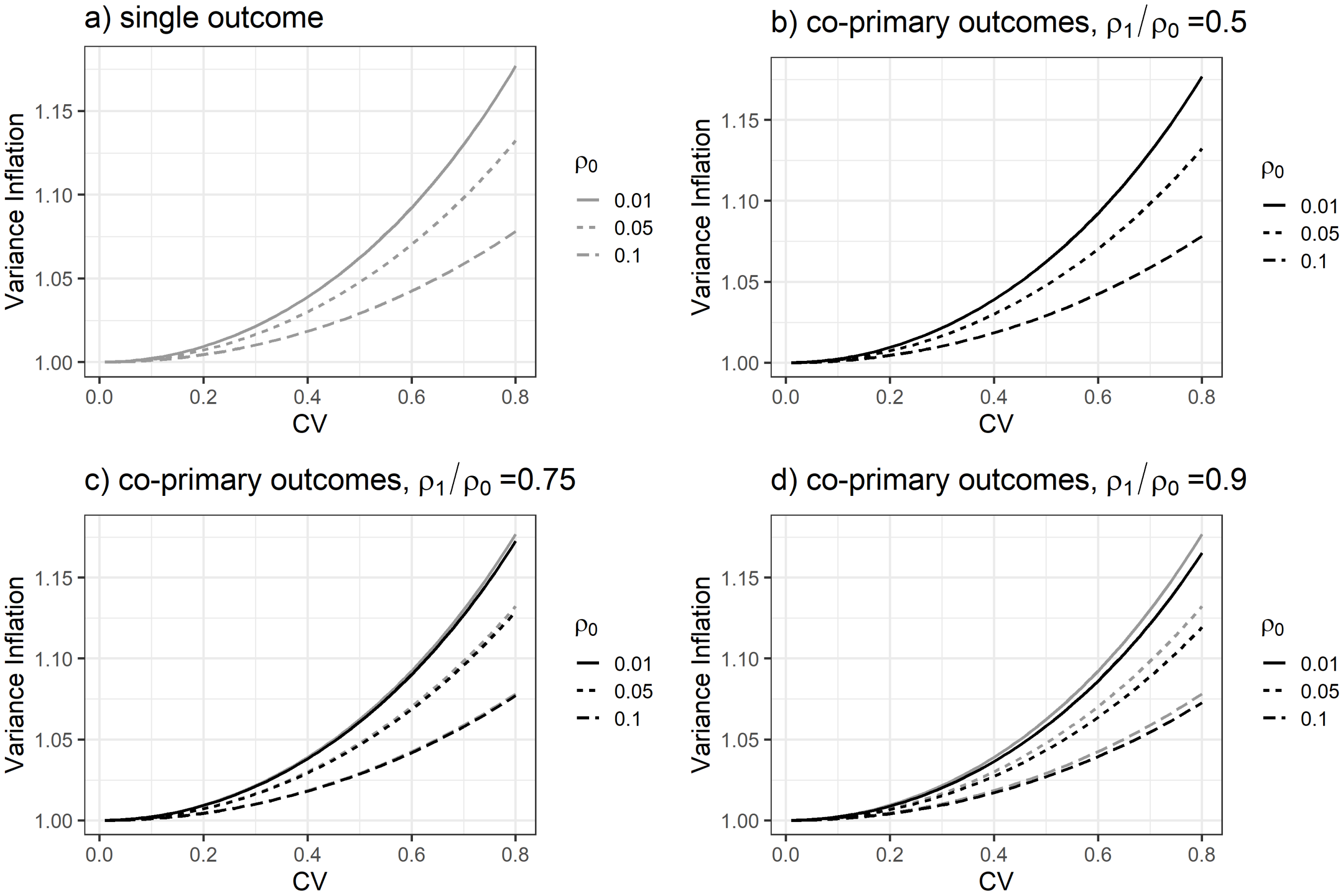

Web Figure 2: Correction factor or variance inflation due to unequal cluster sizes for MLMM and separate LMM analyses of CRTs with co-primary endpoint. (a) Variance inflation for the treatment effect estimator for separate LMM analysis of each endpoint. (b) Variance inflation for the treatment effect estimator for MLMM analysis of two co-primary endpoints when $\rho_1/\rho_0 = 0.5$, $\rho_2 = 0.5$; (c) Variance inflation for the treatment effect estimator for MLMM analysis of two co-primary endpoints when $\rho_1/\rho_0 = 0.75$, $\rho_2 = 0.5$; (d) Variance inflation for the treatment effect estimator for MLMM analysis of two co-primary endpoints when $\rho_1/\rho_0 = 0.9$, $\rho_2 = 0.5$. In (b-d), the gray lines replicate the results in (a) and can facilitate efficiency comparisons between MLMM and separate LMM analyses.

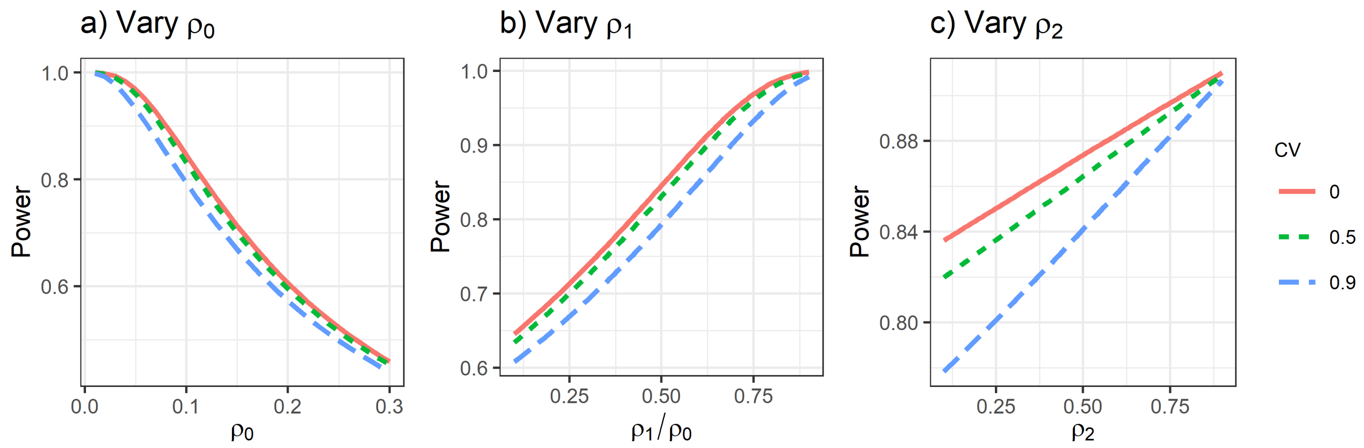

Web Figure 3: Power of the test for treatment effect homogeneity with $K = 2$ co-primary endpoints as a function of (a) endpoint-specific ICC ρ_0, when fixing $\rho_1/\rho_0 = 0.5$ and $\rho_2 = 0.2$; (b) inter-subject cross-endpoint ICC ρ_1 when fixing $\rho_0 = 0.1$ and $\rho_2 = 0.2$; (c) intra-subject ICC ρ_2, when fixing $\rho_0 = 0.1$ and $\rho_1/\rho_0 = 0.5$. All scenarios assume $n = 30$, $\overline{m} = 60$, $\boldsymbol{\beta} = (0.3, 0.7)^T$, $\sigma^2_{yk} = 1$ and equal randomization $\sigma^2_z = 1/4$. All figures assume the block exchangeable correlation structure such that $\rho^k_0 = \rho_0$, $\rho^{kk'}_1 = \rho_1$, $\rho^{kk'}_2 = \rho_2$ for $k \neq k' \in \{1, 2\}$.

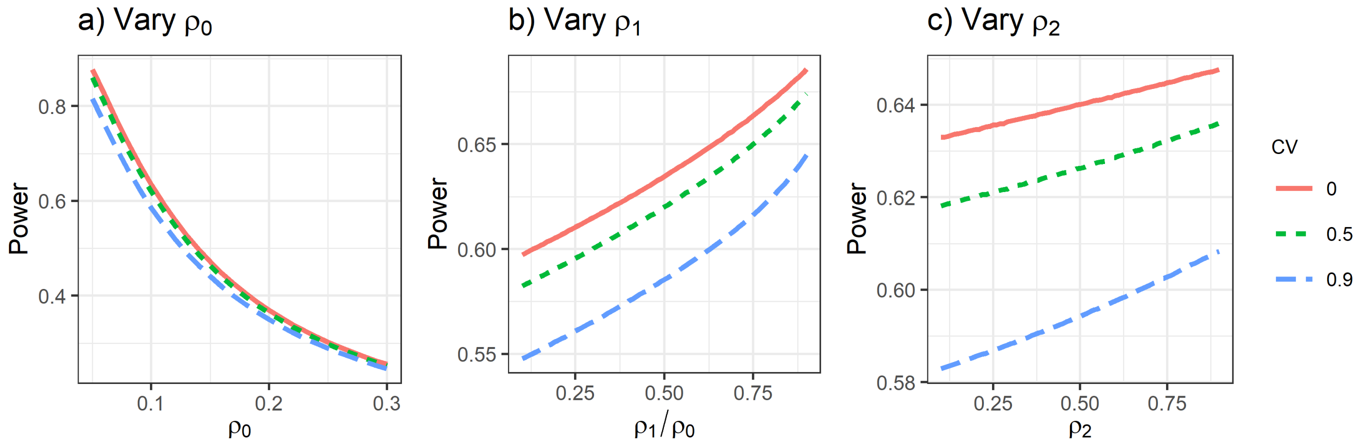

Web Figure 4: Power of the intersection-union test with $K = 2$ co-primary endpoints as a function of (a) endpoint-specific ICC ρ_0, when fixing $\rho_1/\rho_0 = 0.5$ and $\rho_2 = 0.2$; (b) inter-subject cross-endpoint ICC ρ_1 when fixing $\rho_0 = 0.1$ and $\rho_2 = 0.2$; (c) intra-subject ICC ρ_2, when fixing $\rho_0 = 0.1$ and $\rho_1/\rho_0 = 0.5$. All scenarios assume $n = 30$, $\overline{m} = 60$, $\boldsymbol{\beta} = (0.3, 0.3)^T$, $\sigma^2_{yk} = 1$ and equal randomization with $\sigma^2_z = 1/4$. All figures assume the block exchangeable correlation structure such that $\rho_0^k = \rho_0$, $\rho_1^{kk'} = \rho_1$, $\rho_2^{kk'} = \rho_2$ for $k \neq k' \in \{1, 2\}$.

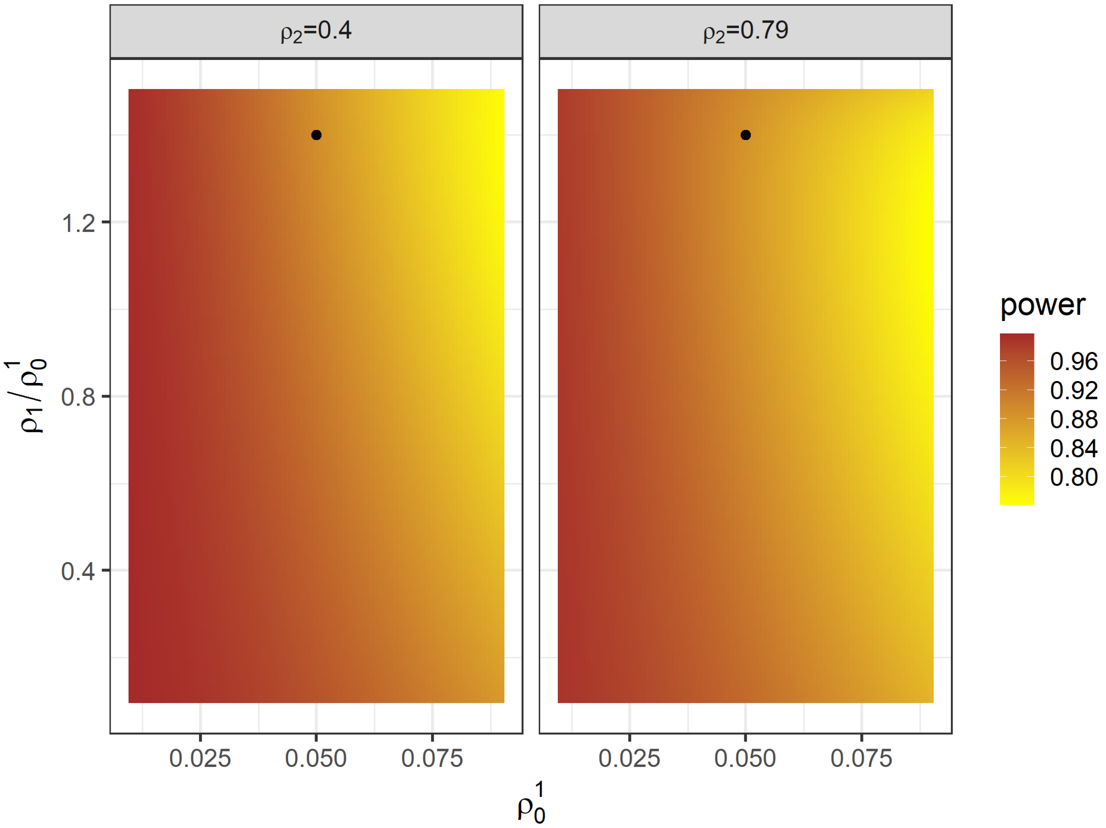

Web Figure 5: Predicted power for the omnibus test with $n = 60$ clusters with varying ICC values as additional sensitivity analysis. The predicted power corresponding to the ICC values estimated from the K-DPP trial is highlighted with a solid black dot.

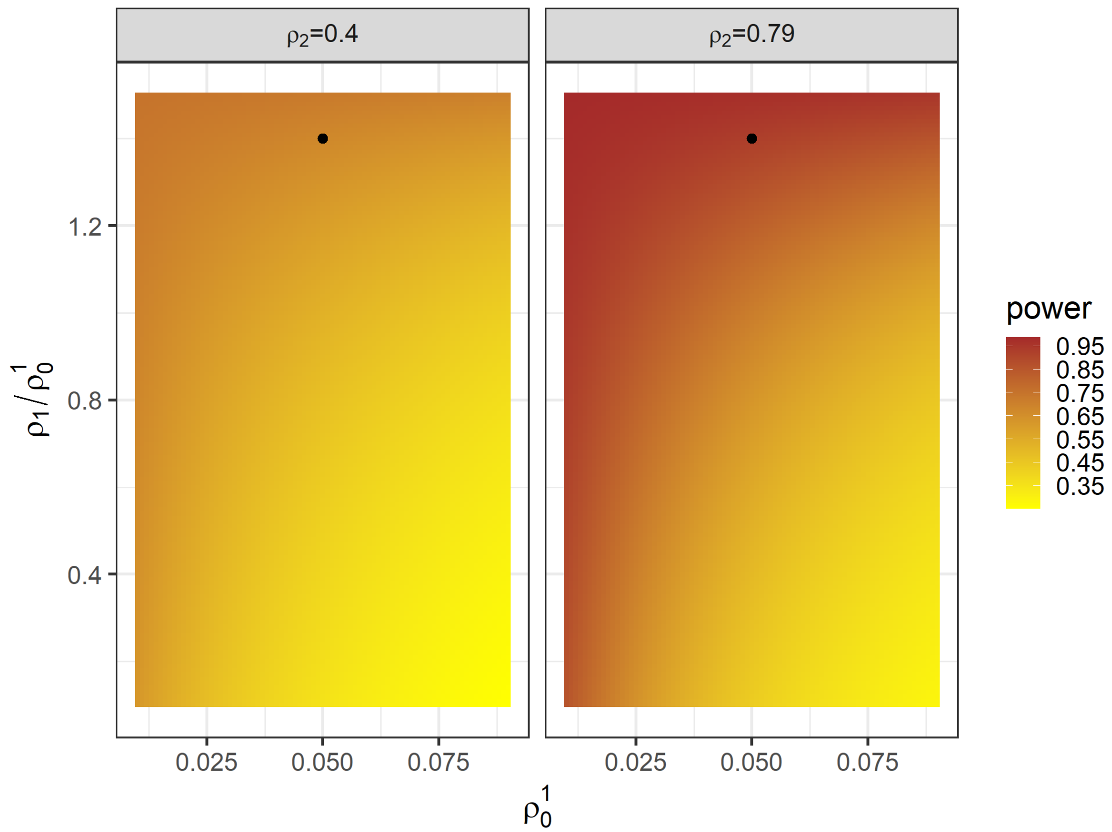

Web Figure 6: Predicted power for the test for treatment effect homogeneity with $n = 60$ clusters with varying ICC values as additional sensitivity analysis. The predicted power corresponding to the ICC values estimated from the K-DPP trial is highlighted with a solid black dot.

Web Appendix H: Web Tables

Web Table 1: A summary of all varied parameters in the simulation study. Notation: $\mathbf{1}_K$ indicates a K-vector of ones.

Parameter	$K = 2$	$K = 3$
$\boldsymbol{\rho}_0$	$\{(0.01, 0.1), (0.05, 0.1)\}$	$\{(0.01, 0.055, 0.1), (0.05, 0.075, 0.1)\}$
$\boldsymbol{\rho}_1$	$\{0.005, 0.025\}$	$\{0.005 \times \mathbf{1}_3, 0.025 \times \mathbf{1}_3\}$
$\boldsymbol{\rho}_2$	$\{0.2, 0.5\}$	$\{0.2 \times \mathbf{1}_3, 0.5 \times \mathbf{1}_3\}$
CV	$\{0, 0.2, 0.4, 0.8\}$	$\{0, 0.2, 0.4, 0.8\}$
$\boldsymbol{\beta}$	$\{(0.3, 0.7), (0.5, 0.7)\}$	$\{(0.3, 0.5, 0.7), (0.5, 0.6, 0.7)\}$

Web Table 2: Estimated required number of clusters n, predicted power ψ, empirical power $\overline{\psi}$, and type I error rate e with $K = 3$, different levels of effect sizes, CV of cluster sizes, ICC values and mean cluster sizes $\overline{m}$.

				$\overline{m} = 60$				$\overline{m} = 80$			
Effect Size	CV	κ	ρ_2	n	ψ	$\overline{\psi}$	e	n	ψ	$\overline{\psi}$	e
(0.3, 0.5, 0.7)	0.0	0.01	0.20	24	0.820	0.859	0.041	24	0.839	0.837	0.041
		0.01	0.50	24	0.822	0.863	0.045	24	0.841	0.840	0.047
		0.05	0.20	30	0.832	0.850	0.049	28	0.820	0.838	0.056
		0.05	0.50	28	0.803	0.805	0.042	28	0.824	0.841	0.055
	0.2	0.01	0.20	24	0.818	0.834	0.038	24	0.837	0.854	0.052
		0.01	0.50	24	0.820	0.831	0.043	24	0.839	0.854	0.059
		0.05	0.20	30	0.830	0.847	0.055	28	0.818	0.834	0.046
		0.05	0.50	30	0.834	0.854	0.055	28	0.822	0.837	0.048
	0.4	0.01	0.20	24	0.810	0.840	0.052	24	0.832	0.859	0.041
		0.01	0.50	24	0.814	0.848	0.055	24	0.834	0.857	0.057
		0.05	0.20	30	0.821	0.833	0.066	28	0.811	0.827	0.060
		0.05	0.50	30	0.825	0.842	0.069	28	0.814	0.829	0.065
	0.8	0.01	0.20	26	0.818	0.822	0.060	24	0.807	0.819	0.045
		0.01	0.50	26	0.826	0.833	0.056	24	0.814	0.824	0.056
		0.05	0.20	32	0.818	0.821	0.044	30	0.817	0.830	0.056
		0.05	0.50	32	0.819	0.834	0.049	30	0.817	0.838	0.059
(0.5, 0.6, 0.7)	0.0	0.01	0.20	22	0.829	0.854	0.042	20	0.801	0.846	0.047
		0.01	0.50	22	0.830	0.857	0.038	20	0.802	0.848	0.043
		0.05	0.20	22	0.806	0.821	0.047	22	0.821	0.842	0.047
		0.05	0.50	22	0.808	0.822	0.051	22	0.823	0.848	0.054
	0.2	0.01	0.20	22	0.827	0.846	0.042	22	0.841	0.849	0.063
		0.01	0.50	22	0.828	0.848	0.044	20	0.800	0.820	0.036
		0.05	0.20	22	0.803	0.835	0.051	22	0.819	0.825	0.065
		0.05	0.50	22	0.806	0.825	0.048	22	0.821	0.826	0.061
	0.4	0.01	0.20	22	0.821	0.832	0.050	22	0.837	0.858	0.054
		0.01	0.50	22	0.823	0.834	0.064	22	0.839	0.860	0.053
		0.05	0.20	24	0.837	0.853	0.053	22	0.814	0.850	0.060
		0.05	0.50	22	0.800	0.817	0.054	22	0.817	0.841	0.062
	0.8	0.01	0.20	24	0.834	0.865	0.044	22	0.818	0.826	0.049
		0.01	0.50	22	0.803	0.821	0.047	22	0.824	0.840	0.053
		0.05	0.20	24	0.811	0.853	0.054	24	0.833	0.837	0.051
		0.05	0.50	24	0.817	0.848	0.052	24	0.838	0.842	0.053

Web Table 3: The predicted power ψ, empirical power $\overline{\psi}$, difference between $\overline{\psi}$ and ψ, and the expected standard error of empirical power $SE(\overline{\psi})$ corresponding to Table 3 with $K = 2$, different levels of effect sizes, CV of cluster sizes, ICC values and mean cluster sizes $\overline{m}$. The boldfaced entries indicate cases where the difference between empirical and predicted power falls within two expected SE of empirical power. The remaining cases demonstrate that empirical power are higher than the formula predictions, suggesting that our power procedure is at most conservative.

				$\overline{m}=60$				$\overline{m}=80$			
Effect Size	CV	κ	ρ_2	ψ	$\overline{\psi}$	$\overline{\psi}-\psi$	$SE(\overline{\psi})$	ψ	$\overline{\psi}$	$\overline{\psi}-\psi$	$SE(\overline{\psi})$
(0.3, 0.7)	0.0	0.01	0.20	0.841	0.854	**0.013**	0.011	0.804	0.823	**0.019**	0.012
		0.01	0.50	0.843	0.855	**0.012**	0.011	0.806	0.822	**0.016**	0.012
		0.05	0.20	0.810	0.815	**0.005**	0.012	0.831	0.847	**0.016**	0.011
		0.05	0.50	0.812	0.828	**0.016**	0.012	0.833	0.840	**0.007**	0.012
	0.2	0.01	0.20	0.838	0.865	0.027	0.011	0.802	0.824	**0.022**	0.012
		0.01	0.50	0.841	0.871	0.030	0.011	0.804	0.826	**0.022**	0.012
		0.05	0.20	0.807	0.835	0.028	0.012	0.829	0.845	**0.016**	0.011
		0.05	0.50	0.809	0.823	**0.014**	0.012	0.830	0.849	**0.019**	0.011
	0.4	0.01	0.20	0.832	0.844	**0.012**	0.011	0.856	0.865	**0.009**	0.011
		0.01	0.50	0.834	0.847	**0.013**	0.011	0.858	0.870	**0.012**	0.011
		0.05	0.20	0.836	0.855	**0.019**	0.011	0.822	0.841	**0.019**	0.012
		0.05	0.50	0.837	0.865	0.028	0.011	0.823	0.833	**0.010**	0.012
	0.8	0.01	0.20	0.800	0.784	**-0.016**	0.013	0.833	0.833	**0.000**	0.012
		0.01	0.50	0.805	0.804	**-0.001**	0.013	0.836	0.841	**0.005**	0.012
		0.05	0.20	0.802	0.810	**0.008**	0.012	0.831	0.867	0.036	0.011
		0.05	0.50	0.832	0.841	**0.009**	0.012	0.828	0.856	0.028	0.011
(0.5, 0.7)	0.0	0.01	0.20	0.814	0.854	0.040	0.011	0.825	0.839	**0.014**	0.012
		0.01	0.50	0.814	0.857	0.043	0.011	0.825	0.839	**0.014**	0.012
		0.05	0.20	0.853	0.870	**0.017**	0.011	0.809	0.826	**0.017**	0.012
		0.05	0.50	0.854	0.874	**0.020**	0.010	0.810	0.829	**0.019**	0.012
	0.2	0.01	0.20	0.813	0.838	0.025	0.012	0.824	0.846	**0.022**	0.011
		0.01	0.50	0.813	0.834	**0.021**	0.012	0.824	0.843	**0.019**	0.012
		0.05	0.20	0.851	0.875	0.024	0.010	0.808	0.832	**0.024**	0.012
		0.05	0.50	0.852	0.882	0.030	0.010	0.809	0.838	0.029	0.012
	0.4	0.01	0.20	0.808	0.829	**0.021**	0.012	0.821	0.863	0.042	0.011
		0.01	0.50	0.809	0.826	**0.017**	0.012	0.821	0.867	0.046	0.011
		0.05	0.20	0.846	0.862	**0.016**	0.011	0.803	0.840	0.037	0.012
		0.05	0.50	0.847	0.859	**0.012**	0.011	0.805	0.845	0.040	0.011
	0.8	0.01	0.20	0.842	0.841	**-0.001**	0.012	0.806	0.854	0.048	0.011
		0.01	0.50	0.847	0.851	**0.004**	0.011	0.810	0.851	0.041	0.011
		0.05	0.20	0.822	0.817	**-0.005**	0.012	0.842	0.857	**0.015**	0.011
		0.05	0.50	0.825	0.820	**-0.005**	0.012	0.845	0.853	**0.008**	0.011

Web Table 4: The predicted power ψ, empirical power $\overline{\psi}$, difference between $\overline{\psi}$ and ψ, and the expected standard error of empirical power $SE(\overline{\psi})$ corresponding to Web Table 2 with $K = 3$, different levels of effect sizes, CV of cluster sizes, ICC values and mean cluster sizes $\overline{m}$. The boldfaced entries indicate cases where the difference between empirical and predicted power falls within two expected SE of empirical power. The remaining cases demonstrate that empirical power are higher than the formula predictions, suggesting that our power procedure is at most conservative.

				$\overline{m} = 60$				$\overline{m} = 80$			
Effect Size	CV	κ	ρ_2	ψ	$\overline{\psi}$	$\overline{\psi} - \psi$	$SE(\overline{\psi})$	ψ	$\overline{\psi}$	$\overline{\psi} - \psi$	$SE(\overline{\psi})$
(0.3, 0.5, 0.7)	0.0	0.01	0.20	0.820	0.859	0.039	0.011	0.839	0.837	**-0.002**	0.012
		0.01	0.50	0.822	0.863	0.041	0.011	0.841	0.840	**-0.001**	0.012
		0.05	0.20	0.832	0.850	**0.018**	0.011	0.820	0.838	**0.018**	0.012
		0.05	0.50	0.803	0.805	**0.002**	0.013	0.824	0.841	**0.017**	0.012
	0.2	0.01	0.20	0.818	0.834	**0.016**	0.012	0.837	0.854	**0.017**	0.011
		0.01	0.50	0.820	0.831	**0.011**	0.012	0.839	0.854	**0.015**	0.011
		0.05	0.20	0.830	0.847	**0.017**	0.011	0.818	0.834	**0.016**	0.012
		0.05	0.50	0.834	0.854	**0.020**	0.011	0.822	0.837	**0.015**	0.012
	0.4	0.01	0.20	0.810	0.840	0.030	0.012	0.832	0.859	0.027	0.011
		0.01	0.50	0.814	0.848	0.034	0.011	0.834	0.857	0.023	0.011
		0.05	0.20	0.821	0.833	**0.012**	0.012	0.811	0.827	**0.016**	0.012
		0.05	0.50	0.825	0.842	**0.017**	0.012	0.814	0.829	**0.015**	0.012
	0.8	0.01	0.20	0.818	0.822	**0.004**	0.012	0.807	0.819	**0.012**	0.012
		0.01	0.50	0.826	0.833	**0.007**	0.012	0.814	0.824	**0.010**	0.012
		0.05	0.20	0.818	0.821	**0.003**	0.012	0.817	0.830	**0.013**	0.012
		0.05	0.50	0.819	0.834	**0.015**	0.012	0.817	0.838	**0.021**	0.012
(0.5, 0.6, 0.7)	0.0	0.01	0.20	0.829	0.854	0.025	0.011	0.801	0.846	0.045	0.011
		0.01	0.50	0.830	0.857	0.027	0.011	0.802	0.848	0.046	0.011
		0.05	0.20	0.806	0.821	**0.015**	0.012	0.821	0.842	**0.021**	0.012
		0.05	0.50	0.808	0.822	**0.014**	0.012	0.823	0.848	0.025	0.011
	0.2	0.01	0.20	0.827	0.846	**0.019**	0.011	0.841	0.849	**0.008**	0.011
		0.01	0.50	0.828	0.848	**0.020**	0.011	0.800	0.820	**0.020**	0.012
		0.05	0.20	0.803	0.835	0.032	0.012	0.819	0.825	**0.006**	0.012
		0.05	0.50	0.806	0.825	**0.019**	0.012	0.821	0.826	**0.005**	0.012
	0.4	0.01	0.20	0.821	0.832	**0.011**	0.012	0.837	0.858	**0.021**	0.011
		0.01	0.50	0.823	0.834	**0.011**	0.012	0.839	0.860	**0.021**	0.011
		0.05	0.20	0.837	0.853	**0.016**	0.011	0.814	0.850	0.036	0.011
		0.05	0.50	0.800	0.817	**0.017**	0.012	0.817	0.841	**0.024**	0.012
	0.8	0.01	0.20	0.834	0.865	0.031	0.011	0.818	0.826	**0.008**	0.012
		0.01	0.50	0.803	0.821	**0.018**	0.012	0.824	0.840	**0.016**	0.012
		0.05	0.20	0.811	0.853	0.042	0.011	0.833	0.837	**0.004**	0.012
		0.05	0.50	0.817	0.848	0.031	0.011	0.838	0.842	**0.004**	0.012

Web Table 5: Bias of variance components (difference between mean of estimates and truth) when $K = 2, \overline{m} = 60$ across different simulation scenarios.

(η, CV, κ, ρ_2, n)	σ^2_{e1}	σ_{e12}	σ^2_{e2}	$\sigma^2_{\phi1}$	$\sigma_{\phi12}$	$\sigma^2_{\phi2}$
(0.3, 0.0, 0.01, 0.2, 16)	-0.003	0.000	-0.001	-0.002	-0.001	-0.027
(0.5, 0.0, 0.01, 0.2, 14)	-0.001	-0.001	-0.007	-0.002	-0.002	-0.033
(0.3, 0.2, 0.01, 0.2, 16)	-0.002	0.000	-0.004	-0.002	-0.001	-0.033
(0.5, 0.2, 0.01, 0.2, 14)	-0.002	-0.001	-0.002	-0.002	-0.002	-0.031
(0.3, 0.4, 0.01, 0.2, 16)	-0.003	0.000	-0.002	-0.002	-0.002	-0.026
(0.5, 0.4, 0.01, 0.2, 14)	-0.003	-0.002	-0.002	-0.002	-0.001	-0.032
(0.3, 0.8, 0.01, 0.2, 16)	-0.003	-0.001	-0.001	-0.002	-0.002	-0.037
(0.5, 0.8, 0.01, 0.2, 16)	-0.003	-0.001	-0.001	-0.002	-0.002	-0.037
(0.3, 0.0, 0.05, 0.2, 22)	-0.002	-0.002	-0.002	-0.006	-0.003	-0.021
(0.5, 0.0, 0.05, 0.2, 16)	-0.002	0.000	-0.001	-0.007	-0.004	-0.027
(0.3, 0.2, 0.05, 0.2, 22)	-0.001	-0.001	-0.005	-0.006	-0.003	-0.021
(0.5, 0.2, 0.05, 0.2, 16)	0.000	0.000	-0.003	-0.008	-0.006	-0.033
(0.3, 0.4, 0.05, 0.2, 24)	-0.002	0.000	-0.002	-0.005	-0.003	-0.017
(0.5, 0.4, 0.05, 0.2, 16)	-0.001	0.000	-0.001	-0.009	-0.005	-0.026
(0.3, 0.8, 0.05, 0.2, 24)	-0.001	0.000	-0.005	-0.006	-0.004	-0.022
(0.5, 0.8, 0.05, 0.2, 16)	-0.002	-0.001	-0.001	-0.008	-0.007	-0.037
(0.3, 0.0, 0.01, 0.5, 16)	-0.002	-0.001	-0.001	-0.002	-0.001	-0.026
(0.5, 0.0, 0.01, 0.5, 14)	-0.002	-0.004	-0.008	-0.002	-0.002	-0.032
(0.3, 0.2, 0.01, 0.5, 16)	-0.002	-0.002	-0.004	-0.002	-0.001	-0.032
(0.5, 0.2, 0.01, 0.5, 14)	-0.003	-0.002	-0.002	-0.002	-0.002	-0.030
(0.3, 0.4, 0.01, 0.5, 16)	-0.002	-0.001	-0.002	-0.002	-0.001	-0.026
(0.5, 0.4, 0.01, 0.5, 14)	-0.003	-0.003	-0.003	-0.002	-0.001	-0.031
(0.3, 0.8, 0.01, 0.5, 16)	-0.003	-0.001	-0.001	-0.002	-0.002	-0.036
(0.5, 0.8, 0.01, 0.5, 16)	-0.003	-0.001	-0.001	-0.002	-0.002	-0.036
(0.3, 0.0, 0.05, 0.5, 22)	-0.003	-0.002	-0.002	-0.006	-0.004	-0.021
(0.5, 0.0, 0.05, 0.5, 16)	-0.001	0.000	-0.001	-0.007	-0.005	-0.027
(0.3, 0.2, 0.05, 0.5, 22)	-0.002	-0.002	-0.005	-0.005	-0.003	-0.021
(0.5, 0.2, 0.05, 0.5, 16)	0.000	-0.001	-0.004	-0.008	-0.007	-0.033
(0.3, 0.4, 0.05, 0.5, 24)	-0.002	-0.001	-0.002	-0.005	-0.003	-0.017
(0.5, 0.4, 0.05, 0.5, 16)	-0.001	0.000	-0.001	-0.009	-0.006	-0.026
(0.3, 0.8, 0.05, 0.5, 26)	0.001	-0.001	-0.004	-0.006	-0.003	-0.015
(0.5, 0.8, 0.05, 0.5, 16)	-0.001	-0.001	-0.001	-0.008	-0.008	-0.037

Web Table 6: Bias of variance components (difference between mean of estimates and truth) when $K = 2, \overline{m} = 80$ across different simulation scenarios.

(η, CV, κ, ρ_2, n)	σ^2_{e1}	σ_{e12}	σ^2_{e2}	$\sigma^2_{\phi 1}$	$\sigma_{\phi 12}$	$\sigma^2_{\phi 2}$
(0.3, 0.0, 0.01, 0.2, 14)	-0.001	-0.002	-0.005	-0.002	0.000	-0.029
(0.5, 0.0, 0.01, 0.2, 14)	-0.001	-0.002	-0.005	-0.002	0.000	-0.029
(0.3, 0.2, 0.01, 0.2, 14)	-0.002	-0.001	-0.002	-0.002	-0.001	-0.033
(0.5, 0.2, 0.01, 0.2, 14)	-0.002	-0.001	-0.002	-0.002	-0.001	-0.033
(0.3, 0.4, 0.01, 0.2, 16)	-0.001	0.000	-0.002	-0.002	-0.002	-0.031
(0.5, 0.4, 0.01, 0.2, 14)	-0.003	-0.002	-0.001	-0.002	-0.002	-0.033
(0.3, 0.8, 0.01, 0.2, 16)	-0.002	-0.001	-0.001	-0.002	-0.002	-0.028
(0.5, 0.8, 0.01, 0.2, 14)	-0.003	-0.001	-0.004	-0.002	-0.001	-0.034
(0.3, 0.0, 0.05, 0.2, 22)	0.000	-0.001	-0.004	-0.005	-0.003	-0.017
(0.5, 0.0, 0.05, 0.2, 14)	0.000	-0.002	-0.005	-0.009	-0.004	-0.029
(0.3, 0.2, 0.05, 0.2, 22)	0.000	-0.002	-0.004	-0.005	-0.002	-0.021
(0.5, 0.2, 0.05, 0.2, 14)	-0.001	0.000	-0.002	-0.008	-0.006	-0.033
(0.3, 0.4, 0.05, 0.2, 22)	0.002	-0.001	-0.004	-0.005	-0.003	-0.018
(0.5, 0.4, 0.05, 0.2, 14)	-0.002	-0.001	-0.001	-0.010	-0.006	-0.033
(0.3, 0.8, 0.05, 0.2, 24)	0.001	-0.001	-0.005	-0.007	-0.005	-0.024
(0.5, 0.8, 0.05, 0.2, 16)	-0.001	-0.001	-0.001	-0.008	-0.006	-0.028
(0.3, 0.0, 0.01, 0.5, 14)	-0.003	-0.004	-0.005	-0.002	0.000	-0.029
(0.5, 0.0, 0.01, 0.5, 14)	-0.003	-0.004	-0.005	-0.002	0.000	-0.029
(0.3, 0.2, 0.01, 0.5, 14)	-0.002	-0.001	-0.002	-0.002	-0.002	-0.032
(0.5, 0.2, 0.01, 0.5, 14)	-0.002	-0.001	-0.002	-0.002	-0.002	-0.032
(0.3, 0.4, 0.01, 0.5, 16)	-0.001	-0.001	-0.002	-0.002	-0.002	-0.030
(0.5, 0.4, 0.01, 0.5, 14)	-0.003	-0.002	-0.002	-0.002	-0.002	-0.032
(0.3, 0.8, 0.01, 0.5, 16)	-0.002	-0.002	-0.001	-0.002	-0.002	-0.027
(0.5, 0.8, 0.01, 0.5, 14)	-0.003	-0.003	-0.005	-0.003	-0.001	-0.033
(0.3, 0.0, 0.05, 0.5, 22)	-0.001	-0.002	-0.003	-0.005	-0.003	-0.016
(0.5, 0.0, 0.05, 0.5, 14)	-0.002	-0.003	-0.005	-0.008	-0.004	-0.030
(0.3, 0.2, 0.05, 0.5, 22)	-0.001	-0.003	-0.003	-0.005	-0.003	-0.021
(0.5, 0.2, 0.05, 0.5, 14)	-0.001	-0.001	-0.002	-0.009	-0.007	-0.033
(0.3, 0.4, 0.05, 0.5, 22)	0.001	-0.002	-0.004	-0.005	-0.003	-0.018
(0.5, 0.4, 0.05, 0.5, 14)	-0.002	-0.001	-0.001	-0.010	-0.006	-0.033
(0.3, 0.8, 0.05, 0.5, 24)	0.000	-0.002	-0.005	-0.007	-0.005	-0.024
(0.5, 0.8, 0.05, 0.5, 16)	-0.001	-0.001	-0.001	-0.008	-0.007	-0.028

Web Table 7: Bias of variance components (difference between mean of estimates and truth) in $\boldsymbol{\Sigma_e}$ when $K = 3, \overline{m} = 60$ across different simulation scenarios.

(η, CV, κ, ρ_2, n)	σ^2_{e1}	$\sigma^2_{e_{12}}$	σ^2_{e2}	$\sigma^2_{e_{31}}$	$\sigma^2_{e_{32}}$	σ^2_{e3}
(0.3, 0.0, 0.01, 0.2, 24)	-0.001	-0.001	-0.003	0.000	-0.001	0.001
(0.5, 0.0, 0.01, 0.2, 22)	0.000	0.000	-0.001	-0.001	0.001	-0.007
(0.3, 0.2, 0.01, 0.2, 24)	0.000	-0.001	-0.002	-0.001	0.001	-0.004
(0.5, 0.2, 0.01, 0.2, 22)	0.001	-0.001	-0.003	-0.002	-0.003	-0.007
(0.3, 0.4, 0.01, 0.2, 24)	0.000	-0.002	-0.002	0.001	0.000	-0.004
(0.5, 0.4, 0.01, 0.2, 22)	0.002	-0.002	-0.001	-0.002	-0.004	-0.007
(0.3, 0.8, 0.01, 0.2, 26)	0.001	0.001	-0.002	-0.001	-0.005	-0.002
(0.5, 0.8, 0.01, 0.2, 24)	0.001	-0.001	-0.004	0.000	-0.004	-0.006
(0.3, 0.0, 0.05, 0.2, 30)	-0.001	0.002	0.001	-0.003	-0.002	-0.006
(0.5, 0.0, 0.05, 0.2, 22)	0.001	0.000	-0.001	-0.001	0.001	-0.007
(0.3, 0.2, 0.05, 0.2, 30)	0.000	-0.002	-0.002	0.000	-0.003	-0.006
(0.5, 0.2, 0.05, 0.2, 22)	0.002	-0.001	-0.002	-0.002	-0.002	-0.007
(0.3, 0.4, 0.05, 0.2, 30)	0.001	-0.001	-0.001	-0.001	-0.003	-0.006
(0.5, 0.4, 0.05, 0.2, 24)	0.001	-0.002	-0.002	0.001	0.000	-0.004
(0.3, 0.8, 0.05, 0.2, 32)	0.000	0.000	0.001	-0.001	-0.003	-0.006
(0.5, 0.8, 0.05, 0.2, 24)	0.002	-0.001	-0.004	0.000	-0.003	-0.006
(0.3, 0.0, 0.01, 0.5, 24)	-0.001	-0.001	-0.003	0.000	-0.001	0.000
(0.5, 0.0, 0.01, 0.5, 22)	-0.001	-0.001	-0.001	-0.003	-0.002	-0.008
(0.3, 0.2, 0.01, 0.5, 24)	-0.001	-0.002	-0.002	-0.002	-0.001	-0.004
(0.5, 0.2, 0.01, 0.5, 22)	-0.001	-0.003	-0.004	-0.004	-0.005	-0.007
(0.3, 0.4, 0.01, 0.5, 24)	0.000	-0.002	-0.002	-0.001	-0.002	-0.005
(0.5, 0.4, 0.01, 0.5, 22)	-0.001	-0.004	-0.004	-0.004	-0.006	-0.007
(0.3, 0.8, 0.01, 0.5, 26)	0.000	-0.001	-0.005	-0.002	-0.005	-0.002
(0.5, 0.8, 0.01, 0.5, 22)	-0.001	-0.003	-0.004	-0.001	-0.005	-0.003
(0.3, 0.0, 0.05, 0.5, 28)	-0.002	-0.001	0.002	-0.002	0.000	-0.002
(0.5, 0.0, 0.05, 0.5, 22)	0.000	-0.001	-0.001	-0.002	-0.001	-0.007
(0.3, 0.2, 0.05, 0.5, 30)	-0.001	-0.003	-0.004	-0.002	-0.004	-0.005
(0.5, 0.2, 0.05, 0.5, 22)	0.000	-0.002	-0.004	-0.003	-0.004	-0.006
(0.3, 0.4, 0.05, 0.5, 30)	-0.001	-0.002	-0.003	-0.003	-0.004	-0.006
(0.5, 0.4, 0.05, 0.5, 22)	0.000	-0.003	-0.003	-0.003	-0.005	-0.006
(0.3, 0.8, 0.05, 0.5, 32)	-0.001	-0.001	-0.002	-0.002	-0.005	-0.006
(0.5, 0.8, 0.05, 0.5, 24)	0.000	-0.002	-0.005	-0.002	-0.005	-0.006

Web Table 8: Bias of variance components (difference between mean of estimates and truth) in $\boldsymbol{\Sigma_e}$ when $K = 3, \overline{m} = 80$ across different simulation scenarios.

(η, CV, κ, ρ_2, n)	σ^2_{e1}	$\sigma^2_{e_{12}}$	σ^2_{e2}	$\sigma^2_{e_{31}}$	$\sigma^2_{e_{32}}$	σ^2_{e3}
(0.3, 0.0, 0.01, 0.2, 24)	0.000	0.001	0.000	0.001	0.001	-0.002
(0.5, 0.0, 0.01, 0.2, 20)	-0.001	-0.001	0.000	-0.001	-0.004	-0.002
(0.3, 0.2, 0.01, 0.2, 24)	0.000	0.000	-0.001	0.001	-0.002	-0.004
(0.5, 0.2, 0.01, 0.2, 22)	0.000	0.001	0.001	0.001	-0.003	-0.007
(0.3, 0.4, 0.01, 0.2, 24)	0.001	0.002	0.000	0.000	-0.003	-0.006
(0.5, 0.4, 0.01, 0.2, 22)	0.000	0.000	0.000	-0.001	-0.005	-0.003
(0.3, 0.8, 0.01, 0.2, 24)	0.000	0.000	-0.002	-0.001	-0.005	-0.005
(0.5, 0.8, 0.01, 0.2, 22)	-0.002	-0.001	-0.001	0.000	-0.005	0.001
(0.3, 0.0, 0.05, 0.2, 28)	-0.001	-0.001	0.001	-0.001	-0.004	-0.002
(0.5, 0.0, 0.05, 0.2, 22)	0.000	-0.002	0.000	0.000	-0.003	-0.005
(0.3, 0.2, 0.05, 0.2, 28)	0.000	0.001	0.002	0.000	-0.001	-0.001
(0.5, 0.2, 0.05, 0.2, 22)	0.001	0.001	0.001	0.001	-0.003	-0.007
(0.3, 0.4, 0.05, 0.2, 28)	-0.001	-0.001	-0.001	-0.002	-0.004	0.002
(0.5, 0.4, 0.05, 0.2, 22)	0.001	0.000	0.001	-0.001	-0.005	-0.004
(0.3, 0.8, 0.05, 0.2, 30)	0.000	0.001	0.003	0.000	0.000	-0.006
(0.5, 0.8, 0.05, 0.2, 24)	0.001	0.000	-0.001	0.000	-0.005	-0.005
(0.3, 0.0, 0.01, 0.5, 24)	0.000	0.001	0.000	0.000	0.000	-0.002
(0.5, 0.0, 0.01, 0.5, 20)	-0.002	-0.002	-0.003	-0.002	-0.005	-0.003
(0.3, 0.2, 0.01, 0.5, 24)	0.000	-0.001	-0.002	-0.001	-0.003	-0.004
(0.5, 0.2, 0.01, 0.5, 20)	-0.001	-0.001	-0.001	0.000	0.000	0.002
(0.3, 0.4, 0.01, 0.5, 24)	0.001	0.000	-0.002	-0.001	-0.005	-0.006
(0.5, 0.4, 0.01, 0.5, 22)	-0.001	-0.002	-0.003	-0.002	-0.006	-0.003
(0.3, 0.8, 0.01, 0.5, 24)	-0.001	-0.002	-0.005	-0.002	-0.006	-0.005
(0.5, 0.8, 0.01, 0.5, 22)	-0.001	-0.002	-0.004	-0.001	-0.004	0.001
(0.3, 0.0, 0.05, 0.5, 28)	-0.001	-0.002	-0.002	-0.001	-0.004	-0.001
(0.5, 0.0, 0.05, 0.5, 22)	-0.001	-0.002	-0.002	-0.001	-0.004	-0.005
(0.3, 0.2, 0.05, 0.5, 28)	0.000	0.000	0.000	0.000	-0.002	-0.001
(0.5, 0.2, 0.05, 0.5, 22)	0.001	0.000	-0.002	-0.001	-0.005	-0.007
(0.3, 0.4, 0.05, 0.5, 28)	-0.002	-0.002	-0.003	-0.001	-0.003	0.003
(0.5, 0.4, 0.05, 0.5, 22)	0.000	-0.002	-0.003	-0.002	-0.005	-0.003
(0.3, 0.8, 0.05, 0.5, 30)	0.000	0.000	0.002	-0.001	-0.002	-0.006
(0.5, 0.8, 0.05, 0.5, 24)	0.000	-0.001	-0.004	-0.002	-0.006	-0.005

Web Table 9: Bias of variance components (difference between mean of estimates and truth) in $\boldsymbol{\Sigma}_{\boldsymbol{\phi}}$ when $K = 3, \overline{m} = 60$ across different simulation scenarios.

(η, CV, κ, ρ_2, n)	$\sigma^2_{\phi_1}$	$\sigma^2_{\phi_{12}}$	$\sigma^2_{\phi_2}$	$\sigma^2_{\phi_{31}}$	$\sigma^2_{\phi_{32}}$	$\sigma^2_{\phi_3}$
(0.3, 0.0, 0.01, 0.2, 24)	-0.001	-0.001	-0.012	0.000	-0.001	-0.031
(0.5, 0.0, 0.01, 0.2, 22)	-0.001	-0.001	-0.012	-0.002	-0.001	-0.031
(0.3, 0.2, 0.01, 0.2, 24)	-0.001	-0.001	-0.011	-0.001	-0.001	-0.034
(0.5, 0.2, 0.01, 0.2, 22)	-0.001	-0.002	-0.013	-0.001	-0.002	-0.033
(0.3, 0.4, 0.01, 0.2, 24)	-0.001	-0.002	-0.014	-0.002	-0.003	-0.031
(0.5, 0.4, 0.01, 0.2, 22)	-0.001	-0.001	-0.012	-0.001	-0.002	-0.037
(0.3, 0.8, 0.01, 0.2, 26)	-0.001	0.000	-0.011	-0.001	0.000	-0.032
(0.5, 0.8, 0.01, 0.2, 24)	-0.002	-0.001	-0.014	-0.001	-0.002	-0.033
(0.3, 0.0, 0.05, 0.2, 30)	-0.004	-0.002	-0.009	-0.005	-0.003	-0.026
(0.5, 0.0, 0.05, 0.2, 22)	-0.006	-0.003	-0.015	-0.005	-0.005	-0.031
(0.3, 0.2, 0.05, 0.2, 30)	-0.005	-0.003	-0.011	-0.005	-0.005	-0.026
(0.5, 0.2, 0.05, 0.2, 22)	-0.005	-0.004	-0.015	-0.004	-0.007	-0.034
(0.3, 0.4, 0.05, 0.2, 30)	-0.004	-0.004	-0.013	-0.005	-0.006	-0.025
(0.5, 0.4, 0.05, 0.2, 24)	-0.006	-0.004	-0.017	-0.005	-0.007	-0.030
(0.3, 0.8, 0.05, 0.2, 32)	-0.005	-0.003	-0.012	-0.004	-0.003	-0.023
(0.5, 0.8, 0.05, 0.2, 24)	-0.007	-0.004	-0.017	-0.004	-0.007	-0.035
(0.3, 0.0, 0.01, 0.5, 24)	-0.001	-0.001	-0.012	-0.001	-0.002	-0.031
(0.5, 0.0, 0.01, 0.5, 22)	-0.001	-0.001	-0.012	-0.002	-0.002	-0.030
(0.3, 0.2, 0.01, 0.5, 24)	-0.001	-0.001	-0.011	-0.001	-0.001	-0.034
(0.5, 0.2, 0.01, 0.5, 22)	-0.001	-0.002	-0.013	-0.001	-0.002	-0.033
(0.3, 0.4, 0.01, 0.5, 24)	-0.001	-0.002	-0.013	-0.002	-0.003	-0.030
(0.5, 0.4, 0.01, 0.5, 22)	-0.001	-0.001	-0.012	-0.001	-0.002	-0.036
(0.3, 0.8, 0.01, 0.5, 26)	-0.001	0.000	-0.010	-0.001	-0.001	-0.030
(0.5, 0.8, 0.01, 0.5, 22)	-0.002	-0.001	-0.016	-0.001	-0.001	-0.030
(0.3, 0.0, 0.05, 0.5, 28)	-0.004	-0.004	-0.015	-0.004	-0.007	-0.030
(0.5, 0.0, 0.05, 0.5, 22)	-0.006	-0.004	-0.015	-0.006	-0.007	-0.031
(0.3, 0.2, 0.05, 0.5, 30)	-0.005	-0.003	-0.011	-0.005	-0.006	-0.026
(0.5, 0.2, 0.05, 0.5, 22)	-0.005	-0.005	-0.016	-0.004	-0.008	-0.034
(0.3, 0.4, 0.05, 0.5, 30)	-0.004	-0.004	-0.012	-0.005	-0.007	-0.025
(0.5, 0.4, 0.05, 0.5, 22)	-0.006	-0.004	-0.016	-0.006	-0.009	-0.036
(0.3, 0.8, 0.05, 0.5, 32)	-0.005	-0.004	-0.012	-0.004	-0.004	-0.023
(0.5, 0.8, 0.05, 0.5, 24)	-0.007	-0.005	-0.018	-0.006	-0.008	-0.034

Web Table 10: Bias of variance components (difference between mean of estimates and truth) in $\mathbf{\Sigma}_{\phi}$ when $K = 3, \overline{m} = 80$ across different simulation scenarios.

(η, CV, κ, ρ_2, n)	$\sigma^2_{\phi_1}$	$\sigma^2_{\phi_{12}}$	$\sigma^2_{\phi_2}$	$\sigma^2_{\phi_{31}}$	$\sigma^2_{\phi_{32}}$	$\sigma^2_{\phi_3}$
(0.3, 0.0, 0.01, 0.2, 24)	-0.001	-0.001	-0.011	-0.001	-0.002	-0.024
(0.5, 0.0, 0.01, 0.2, 20)	-0.002	-0.001	-0.013	-0.001	-0.002	-0.035
(0.3, 0.2, 0.01, 0.2, 24)	-0.001	-0.002	-0.013	-0.001	-0.002	-0.031
(0.5, 0.2, 0.01, 0.2, 22)	-0.002	-0.002	-0.014	-0.001	-0.002	-0.026
(0.3, 0.4, 0.01, 0.2, 24)	-0.002	-0.001	-0.013	-0.001	-0.002	-0.026
(0.5, 0.4, 0.01, 0.2, 22)	-0.001	-0.001	-0.012	-0.001	-0.004	-0.031
(0.3, 0.8, 0.01, 0.2, 24)	-0.002	-0.001	-0.011	-0.001	-0.001	-0.034
(0.5, 0.8, 0.01, 0.2, 22)	-0.002	-0.001	-0.011	-0.001	-0.002	-0.036
(0.3, 0.0, 0.05, 0.2, 28)	-0.005	-0.003	-0.013	-0.004	-0.006	-0.023
(0.5, 0.0, 0.05, 0.2, 22)	-0.006	-0.003	-0.014	-0.004	-0.002	-0.032
(0.3, 0.2, 0.05, 0.2, 28)	-0.004	-0.001	-0.011	-0.002	-0.004	-0.025
(0.5, 0.2, 0.05, 0.2, 22)	-0.006	-0.005	-0.017	-0.004	-0.005	-0.025
(0.3, 0.4, 0.05, 0.2, 28)	-0.004	-0.002	-0.012	-0.003	-0.006	-0.026
(0.5, 0.4, 0.05, 0.2, 22)	-0.005	-0.004	-0.017	-0.003	-0.009	-0.031
(0.3, 0.8, 0.05, 0.2, 30)	-0.005	-0.002	-0.012	-0.002	-0.002	-0.023
(0.5, 0.8, 0.05, 0.2, 24)	-0.006	-0.002	-0.015	-0.004	-0.006	-0.034
(0.3, 0.0, 0.01, 0.5, 24)	-0.001	-0.001	-0.011	-0.001	-0.002	-0.023
(0.5, 0.0, 0.01, 0.5, 20)	-0.002	0.000	-0.013	-0.001	-0.003	-0.035
(0.3, 0.2, 0.01, 0.5, 24)	-0.001	-0.002	-0.013	-0.001	-0.003	-0.031
(0.5, 0.2, 0.01, 0.5, 20)	-0.002	0.000	-0.012	-0.002	-0.002	-0.039
(0.3, 0.4, 0.01, 0.5, 24)	-0.002	-0.001	-0.012	-0.001	-0.002	-0.026
(0.5, 0.4, 0.01, 0.5, 22)	-0.002	-0.001	-0.012	-0.001	-0.004	-0.031
(0.3, 0.8, 0.01, 0.5, 24)	-0.002	-0.001	-0.010	-0.001	-0.002	-0.033
(0.5, 0.8, 0.01, 0.5, 22)	-0.002	-0.001	-0.011	-0.001	-0.003	-0.034
(0.3, 0.0, 0.05, 0.5, 28)	-0.004	-0.003	-0.013	-0.004	-0.007	-0.023
(0.5, 0.0, 0.05, 0.5, 22)	-0.006	-0.004	-0.015	-0.004	-0.004	-0.032
(0.3, 0.2, 0.05, 0.5, 28)	-0.003	-0.001	-0.011	-0.002	-0.005	-0.025
(0.5, 0.2, 0.05, 0.5, 22)	-0.006	-0.005	-0.017	-0.004	-0.006	-0.026
(0.3, 0.4, 0.05, 0.5, 28)	-0.004	-0.003	-0.012	-0.004	-0.006	-0.026
(0.5, 0.4, 0.05, 0.5, 22)	-0.005	-0.004	-0.017	-0.003	-0.009	-0.031
(0.3, 0.8, 0.05, 0.5, 30)	-0.005	-0.003	-0.012	-0.002	-0.003	-0.023
(0.5, 0.8, 0.05, 0.5, 24)	-0.006	-0.003	-0.014	-0.005	-0.008	-0.034

References

Beilina, L., Karchevskii, E., and Karchevskii, M. (2017). *Numerical Linear Algebra: Theory and Applications.* Springer.

Li, D., Cao, J., and Zhang, S. (2020). Power analysis for cluster randomized trials with multiple binary co-primary endpoints. *Biometrics* **76,** 1064–1074.

Li, F., Turner, E. L., and Preisser, J. S. (2018). Sample size determination for GEE analyses of stepped wedge cluster randomized trials. *Biometrics* **74,** 1450–1458.

Tong, Y. L. (2014). *Probability inequalities in multivariate distributions.* Academic Press.
